# Single-molecule characterization of SV40 replisome and novel factors: human FPC and Mcm10

**DOI:** 10.1093/nar/gkae565

**Published:** 2024-07-05

**Authors:** Yujing Ouyang, Amani Al-Amodi, Muhammad Tehseen, Lubna Alhudhali, Afnan Shirbini, Masateru Takahashi, Vlad-Stefan Raducanu, Gang Yi, Ammar Usman Danazumi, Alfredo De Biasio, Samir M Hamdan

**Affiliations:** Bioscience Program, Division of Biological and Environmental Sciences and Engineering, King Abdullah University of Science and Technology, Thuwal 23955, Saudi Arabia; Bioscience Program, Division of Biological and Environmental Sciences and Engineering, King Abdullah University of Science and Technology, Thuwal 23955, Saudi Arabia; Bioscience Program, Division of Biological and Environmental Sciences and Engineering, King Abdullah University of Science and Technology, Thuwal 23955, Saudi Arabia; Bioscience Program, Division of Biological and Environmental Sciences and Engineering, King Abdullah University of Science and Technology, Thuwal 23955, Saudi Arabia; Bioscience Program, Division of Biological and Environmental Sciences and Engineering, King Abdullah University of Science and Technology, Thuwal 23955, Saudi Arabia; Bioscience Program, Division of Biological and Environmental Sciences and Engineering, King Abdullah University of Science and Technology, Thuwal 23955, Saudi Arabia; Bioscience Program, Division of Biological and Environmental Sciences and Engineering, King Abdullah University of Science and Technology, Thuwal 23955, Saudi Arabia; Bioscience Program, Division of Biological and Environmental Sciences and Engineering, King Abdullah University of Science and Technology, Thuwal 23955, Saudi Arabia; Bioscience Program, Division of Biological and Environmental Sciences and Engineering, King Abdullah University of Science and Technology, Thuwal 23955, Saudi Arabia; Bioscience Program, Division of Biological and Environmental Sciences and Engineering, King Abdullah University of Science and Technology, Thuwal 23955, Saudi Arabia; Bioscience Program, Division of Biological and Environmental Sciences and Engineering, King Abdullah University of Science and Technology, Thuwal 23955, Saudi Arabia

## Abstract

The simian virus 40 (SV40) replisome only encodes for its helicase; large T-antigen (L-Tag), while relying on the host for the remaining proteins, making it an intriguing model system. Despite being one of the earliest reconstituted eukaryotic systems, the interactions coordinating its activities and the identification of new factors remain largely unexplored. Herein, we *in vitro* reconstituted the SV40 replisome activities at the single-molecule level, including DNA unwinding by L-Tag and the single-stranded DNA-binding protein Replication Protein A (RPA), primer extension by DNA polymerase δ, and their concerted leading-strand synthesis. We show that RPA stimulates the processivity of L-Tag without altering its rate and that DNA polymerase δ forms a stable complex with L-Tag during leading-strand synthesis. Furthermore, similar to human and budding yeast Cdc45–MCM–GINS helicase, L-Tag uses the fork protection complex (FPC) and the mini-chromosome maintenance protein 10 (Mcm10) during synthesis. Hereby, we demonstrate that FPC increases this rate, and both FPC and Mcm10 increase the processivity by stabilizing stalled replisomes and increasing their chances of restarting synthesis. The detailed kinetics and novel factors of the SV40 replisome establish it as a closer mimic of the host replisome and expand its application as a model replication system.

## Graphical Abstract

**Graphical Abstract:**
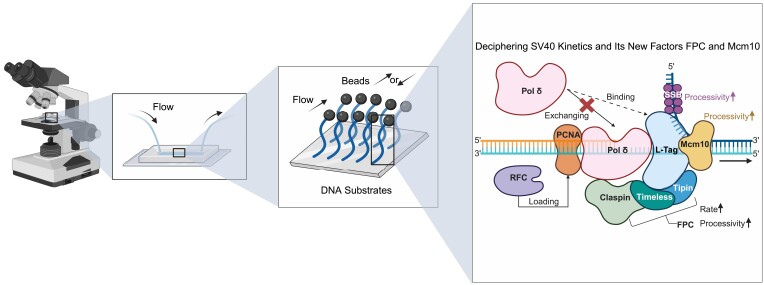

## Introduction

Simian virus 40 (SV40) is a circular double-stranded DNA (dsDNA) virus that can infect humans, monkeys, and other primates. The genome's early genetic region encodes the large T antigen (L-Tag) helicase, which binds to and melts the viral replication origin as a double-hexamer before initiating dsDNA unwinding as a hexamer (1). The L-Tag recruits the DNA polymerase α-primase complex (Pol α-primase) and the single-stranded DNA (ssDNA)-binding protein (SSB) Replication Protein A (RPA) to synthesize primers required to initiate leading- and lagging-strand synthesis (2). Replication Factor C (RFC) recruits the Proliferating Cell Nuclear Antigen (PCNA) to the primer–template for use as a processivity factor by the replicative DNA polymerase δ (Pol δ). Unlike the host replisome, in which DNA Polymerase ϵ (Pol ϵ) replicates the leading strand, and Pol δ replicates the lagging strand, SV40 requires only Pol δ to replicate both strands (3–9). Although the SV40 replisome has been biochemically reconstituted and characterized using purified L-Tag and cell extract mixtures (10,11), few studies have focused on its kinetics and the interactions that coordinate its various activities and communicate with the host histones. Furthermore, the coupling of SV40 replication with the host cell cycle, cellular DNA damage response, and SV40 genomic DNA repair mechanisms are largely unknown. The SV40 replisome's simplicity and reliance on the host proteins make it an attractive model system for reconstitution and characterization to investigate these questions.

Yeast and human replisomes have been reconstituted from purified proteins and have shown many conserved activities (12–17). Both replisomes require several proteins compared to the SV40 replisome, including the chromosomal transmission fidelity 4 (Ctf4 in yeast)/acidic nucleoplasmic DNA-binding protein (AND-1 in humans), bridging the Cdc45–MCM–GINS (CMG) helicase with Pol α-primase (18–25). In humans, PCNA utilizes an alternative clamp-loading mechanism on the leading strand, where Ctf18-RFC and Pol ϵ participate (26). In addition, CMG in both yeast and humans requires a fork protection complex (FPC) composed of Claspin, TIMELESS, Tipin (MTC complex in yeast composed of Mrc1, Tof1, and Csm3), and mini-chromosome maintenance protein 10 (Mcm10) to support the *in vivo* rates of DNA synthesis (13,27–30). FPC appears to function when a replication fork is challenged by DNA damage or barriers. Claspin in FPC stabilizes the DNA replication fork, mediates DNA replication checkpoint activation, and may as well participate in apoptosis, viral infections, and nongenotoxic stress responses (31). TIMELESS and Tipin in FPC also stabilize the DNA replication fork, and participate in cell cycle checkpoint control and chromosome cohesion (32). Mcm10 binds to the N-face of CMG exposed to the fork junction and activates DNA strand annealing to prevent fork regression (33). Additionally, Mcm10 plays a versatile role at the replication fork, including CMG activation during DNA replication initiation, DNA synthesis rate enhancement through CMG binding, and interaction with Pol α-primase and PCNA (34,35).

Several studies have employed single-molecule and bulk-phase assays to characterize the modulation of helicase activity by other replisome proteins. Both *Drosophila melanogaster* CMG (*Dm*CMG) and L-Tag unwind dsDNA as 3′–5′ ssDNA translocases via steric exclusion at 0.1–0.5 and 1–2 bp/s, respectively (36,37). Human RPA stimulates *Dm*CMG unwinding at approximately 5 bp/s (37–47); however, its effect on the L-Tag's rate remains inconclusive but is suggested to have up to 3-fold stimulation (41). The effect of coating the unwound strands with *Escherichia coli* SSB on CMG processivity does not seem critical because *Dm*CMG alone has a similar unwinding processivity to that of yeast CMG in the presence of *E. coli* SSB (12,37). The effect of RPA on DNA unwinding processivity by L-Tag has not been characterized. However, the rates of DNA synthesis by a SV40 replisome reconstituted from L-Tag and Hela cell extracts and a yeast replisome reconstituted from purified proteins were approximately 3 and 20 bp/s, respectively (41,48). This suggests that the rate of L-Tag is modulated modestly within the replisome, whereas the yeast CMG rate is enhanced significantly, mainly driven by the yeast CMG accessory proteins Mcm10 and MTC, which increase the CMG’s DNA unwinding rate and processivity (12). The human replisome, which has been fully reconstituted from purified proteins, displays kinetics similar to those of the reconstituted yeast replisome (49) and utilizes FPC and Mcm10 (17). However, it remains unclear how replisome proteins affect dsDNA unwinding by human CMG helicase.

The stability of helicases and their accessory proteins and polymerases at the fork has also been investigated. In yeast, the MTC complex and Mcm10 are required in excess to sustain the rates measured *in vivo*, suggesting that they do not form a stable complex (12). The cryo-EM structure of the yeast fork protection complex, the Csm3/Tof1 components of MTC, shows them bound to the front of the CMG, gripping the duplex DNA, leading to stabilizing the replisome (14). In yeast, excess Pol ϵ and Pol δ in the solution can exchange with the replisome-associated Pol ϵ and Pol δ, demonstrating some dynamicity. However, this exchange kinetics is very slow, with one polymerase molecule exchanged every ∼10 min, suggesting that the replisome-associated Pol δ and Pol ϵ are highly stable (48). In contrast, yeast CMG is not exchanged during synthesis, which is consistent with its role as an anchor for the replisome (48,50–53). However, the stability of human CMG, Pol δ, and Pol ϵ within the replisome remains uncharacterized.

In contrast to the kinetics described for human and yeast replisomes, it is unclear whether L-Tag forms a stable complex with Pol δ and why L-Tag prefers Pol δ to replicate both leading and lagging strands. Additionally, it is unclear whether L-Tag helicase activity is influenced by RPA and whether L-Tag utilizes other accessory proteins. Claspin physically interacts with L-Tag (54) and is required for efficient herpes simplex virus 1 replication (55). Although Mcm10 does not colocalize with L-Tag *in vivo*, host Pol α-primase colocalizes with L-Tag, which binds Mcm10 in turn (56). Finally, the lifetimes, rates, and processivities of the individual and concerted activities of the SV40 replisome are unknown, obstructing the comparison of its mechanism to that of the host replisome and its use as a model replication system.

Here, we aimed to bridge this knowledge gap and advance our understanding of the SV40 replisome. Using single-molecule flow-stretching assays, we systematically reconstituted primer extension by human Pol δ–PCNA, DNA unwinding by L-Tag in the absence and presence of human RPA, and SV40 leading-strand synthesis. We demonstrated that Pol δ–PCNA synthesized DNA at a mean rate of 240 nt/s with a lifetime of only 2 s. In contrast, L-Tag unwound the DNA at a mean rate of 1 bp/s, and RPA increased L-Tag processivity from a statistically unreliable readout to 0.8 kb without influencing its rate of DNA unwinding. Pol δ–PCNA formed a stable complex with L-Tag to synthesize DNA at a mean rate of 5 bp/s with a mean lifetime of 3 min. Remarkably, the intrinsic processivity of the leading-strand complex suggests that Pol δ does not undergo dynamic exchange during the intrinsic processivity of leading-strand synthesis. Additionally, we established FPC and Mcm10 as new components at the SV40 fork and showed that both FPC and Mcm10 enhance the processivity of leading-strand synthesis by stabilizing the stalled replisome and increasing the chance of restarting DNA synthesis. FPC also increased the rate of leading-strand synthesis through continuous engagement at the fork.

## Materials and methods

### Protein expression and purification

*Plasmid construction*. Human full-length Mcm10 (accession no. Q7L590) N-terminal strept- and 8X histidine-tagged insect cell-optimized sequences were cloned into pFastBac1 using GenScript. The MultiBac™ expression system (Geneva Biotech, Geneva, Switzerland) was used to express human FPC. Insect cell-optimized sequences of human full-length FPC: N-terminus 6X histidine-tagged Claspin (accession no. Q9HAW4), TIMELESS (accession no. Q9UNS1) and C-terminus strept-tagged Tipin (accession no. Q9BVW5) were amplified and cloned into pACEBac1 (Gent^+^), pIDS (Spect^+^) and pIDC (Cm^+^), respectively, using the Gibson assembly protocol. D515V, D602A, D757A, and E795A were mutated in the p125 subunit of wild-type Pol δ (WT Pol δ) (57) using polymerase chain reaction (PCR) to generate a polymerase-and-exonuclease-deficient mutant (hereafter referred to as Pol δ polymerase-deficient mutant). The human RPA clone (pET11d-tRPA) was a generous gift from Professor Marc S. Wold (University of Iowa College of Medicine, Iowa City, IO, USA). The construction of WT Pol δ, WT-PCNA and ΔN-RFC plasmids has been already described previously (57). The full-length L-Tag N-terminally double 6X histidine-tagged insect cell-optimized sequence was cloned into pFastBac1 using GenScript. Finally, single transfer vectors with different subunit assemblies of the Pol δ polymerase-deficient mutant and FPC were generated using *cre* recombinase, according to the MultiBac™ expression system user manual. Finally, Mcm10, WT Pol δ, Pol δ polymerase-deficient mutant, and FPC recombinant transfer vectors were introduced into MultiBac baculoviral DNA in DH10MultiBac™, and bacmid DNA for each plasmid was isolated separately. DNA sequences encoding *E. coli* SSB (amino acids 2–178) and Gibson assembly cloning primers were custom-synthesized by Integrated DNA Technologies (IDT, Coralville, IO, USA). The gene sequence encoding *E. coli* SSB was cloned via Gibson assembly into a modified pE-SUMO-pro expression vector (Lifesensors, Malvern, PA, USA) harboring an N-terminal double His6-Tag followed by the SUMO fusion protein.

*Baculovirus preparation*. To prepare baculovirus, bacmid DNA of WT Pol δ, Pol δ polymerase-deficient mutant, L-Tag, and FPC were transfected separately into Sf9 cells using FuGENE® HD (Promega, Madison, WI, USA), according to the manufacturer's instructions. The supernatant was used as the P1 viral stock and amplified to obtain the P2 viral stock. The P2 viral stock was further amplified for large-scale expression to obtain the P3 viral stock.

*Pol δ and Pol δ mutant*. Human WT Pol δ, Pol δ polymerase-deficient mutant was expressed and purified as described previously (57). Briefly, a 4 L suspension culture of Sf9 cells at 2 × 10^6^ cells/ml was infected with P3 viral stock for 68–72 h. WT Pol δ and Pol δ polymerase-deficient mutant clear lysate were loaded separately on a HisTrap, and bound protein was eluted with imidazole buffer and 50 mM NaCl. Fractions containing proteins were loaded on a Mono Q column, and size exclusion chromatography was performed on HiLoad 16/600 Superdex 200 pg (Cytiva, Marlborough, MA, USA). Protein fractions were pooled, flash-frozen, and stored at −80°C.

*Mcm10 and FPC*. Mcm10 and FPC were expressed by transfecting 4 l of Sf9 suspension culture at 2 × 10^6^ cells/ml with P3 virus for 48 and 72 h, respectively. Cells were harvested by centrifugation at 5500 × *g* for 15 min. The cell pellets were resuspended in 200 ml of lysis buffer (50 mM Tris–HCl (pH 7.5 for Mcm10 and pH 8.0 for FPC), 500 mM NaCl, 20 mM imidazole, 5 mM β-mercaptoethanol, 0.1% NP-40, 1 mM PMSF, 5% glycerol, and EDTA-free protease inhibitor cocktail tablet/50 ml (Roche, Hertfordshire, UK)). Cells were sonicated, and debris was removed by centrifugation at 95 834 × *g* for 1 h at 4 °C. The supernatant was directly loaded onto a HisTrap HP 5 ml affinity column (Cytiva) equilibrated with buffer A (50 mM Tris–HCl (pH 7.5 for Mcm10 and pH 8.0 for FPC), 500 mM NaCl, 20 mM imidazole, 5 mM β-mercaptoethanol, and 5% glycerol). After loading, the column was washed with 50 ml of buffer A, and the bound fractions were eluted by gradient with 50 ml of buffer B (50 mM Tris–HCl (pH 7.5 for Mcm10 and pH 8.0 for FPC), NaCl (300 mM for Mcm10 and 400 mM for FPC), 500 mM imidazole, 5 mM β-mercaptoethanol, and 5% glycerol). Fractions that contain Mcm10 or FPC were combined and loaded onto StrepTrap XT 5 ml (Cytiva) column pre-equilibrated with buffer C (100 mM Tris–HCl (pH 8.0), 300 mM NaCl, 5 mM β-mercaptoethanol, and glycerol (5% for Mcm10 and 10% for FPC)). After loading, the column was extensively washed with buffer C. The Mcm10 or FPC was eluted with 25 ml of buffer D (100 mM Tris–HCl (pH 8.0), 300 mM NaCl, 50 mM biotin, 5 mM β-mercaptoethanol, and 5% glycerol). The fractions containing Mcm10 or FPC were combined. The combined fractions were incubated overnight with TEV protease to remove tags. The fractions were concentrated to 1 ml and loaded onto HiLoad 16/600 Superdex 200 pg pre-equilibrated with gel filtration buffer (50 mM Tris–HCl (pH 7.5 for Mcm10 and pH 8.0 for FPC), NaCl (200 mM for Mcm10 and 300 mM for FPC), 1 mM DTT, and 10% glycerol). Protein fractions were pooled, flash-frozen, and stored at −80°C.

*L-Tag*. L-Tag was expressed by transfecting 4 L of the Sf9 suspension culture at 2 × 10^6^ cells/ml density with the L-Tag P3 virus. After 48 h, the cells were harvested by centrifugation at 5500 × *g* for 10 min and resuspended in lysis buffer (20 mM Tris–HCl (pH 8.0), 50 mM imidazole, 500 mM KCl, 1 mM dithiothreitol, 5% (v/v) glycerol, and EDTA-free protease inhibitor cocktail tablet/50 ml). The cell lysate was clarified by centrifugation at 95 834 × *g* for 1 h at 4°C. The supernatant was directly loaded onto a 5 ml Histrap affinity column pre-equilibrated with buffer A (20 mM Tris–HCl (pH 8.0), 50 mM imidazole, 500 mM KCl, 1 mM DTT and 10% glycerol). The loaded column was washed with 50 ml of buffer A containing 50 mM imidazole, followed by 50 ml of buffer A containing 100 mM imidazole, to remove the nonspecific binding of the protein to the column. Finally, the protein was eluted with a 20 ml gradient to 500 mM imidazole using buffer B containing low salt (20 mM Tris–HCl (pH 8.0), 500 mM imidazole, 250 mM KCl, 1 mM DTT and 10% glycerol). The pooled fractions of L-Tag were treated with TEV protease to remove the His-tag and dialyzed overnight in dialysis buffer (20 mM Tris–HCl (pH 8.0), 100 mM KCl, 1 mM DTT and 10% glycerol). The cleaved protein was then loaded onto Histrap 1 ml affinity column (Cytiva) pre-equilibrated with buffer C (20 mM Tris–HCl (pH 8.0), 250 mM KCl, 1 mM DTT and 10% glycerol). The loaded column was then washed with 6 ml of buffer D, followed by a 6 ml linear gradient of buffer B. Flow-through fractions containing L-Tag were concentrated and loaded onto a HiLoad 16/600 Superdex 200 pg pre-equilibrated with gel filtration buffer (20 mM Tris–HCl (pH 8.0), 100 mM KCl, 1 mM DTT and 10% glycerol). The collected protein was flash-frozen and stored at −80°C.

*PCNA*. Human PCNA was expressed and purified as described previously (57). Briefly, BL21 (DE3) cells containing the PCNA plasmid were grown in 2YT media with ampicillin at 37°C. The protein expression was induced with 0.5 mM isopropyl β-d-1-thiogalactopyranoside (IPTG) when OD_600_ reached 1.2 and incubated further for 19 h at 16°C. The cells were harvested by centrifugation and lysed by sonication. The supernatant was purified using HisTrap HP 5 ml affinity column, followed by a HiTrap Q column, an anion exchanger (Cytiva), and size-exclusion chromatography on a HiLoad 16/600 Superdex 200 pg. The collected fractions were flash-frozen and stored at −80°C.

*ΔN-RFC*. Human N-terminal truncated RFCs (ΔN-RFC) were expressed and purified as described previously (57). Briefly, two plasmids: p14A-ΔN-RFC1/4/2 and pCDFK-RFC5/3, were co-transformed into BL21 (DE3) *E. coli* cells. The cells were grown in a TB medium containing both antibiotics (Kan + Amp) at 20°C to an OD_600_ of 0.8. Protein expression was induced with 0.1 mM IPTG and incubated further for 24 h at 15°C. The cells were collected by centrifugation, lysed using lysozyme, and sonicated. The supernatant was first loaded onto HisTrap HP 5 ml affinity chromatography column, followed by Hitrap Q and size-exclusion chromatography on a HiLoad 16/600 Superdex 200 pg. The purified protein was flash-frozen and stored at −80°C.

*RPA*. Human RPA was expressed and purified as described previously (57). Briefly, the cloned plasmid was transfected into BL21 (DE3) *E. coli*. The cells were grown in 2YT media at 37°C to an OD_600_ of 0.7, and protein expression was induced with 0.5 mM IPTG and further incubated for 4–6 h at 37°C. The cells were collected by centrifugation, lysed with lysozyme, and sonicated. The supernatant was loaded onto HisTrap HP 5 ml column, followed by the HiTrap Blue affinity column (Cytiva). RPA fractions containing all subunits were concentrated and loaded onto the HiLoad 16/600 Superdex 200 pg column. RPA protein fractions were flash-frozen and stored at –80°C.

*E. coli SSB*. *E. coli* SSB was purified as described previously (58). The SSB expression clone was used for the recombinant expression of SSB in BL21 (DE3) *E. coli*. The transformed cells were plated on Kan^+^ selection LB agar (VWR) media. A single colony was inoculated in LB media supplemented with 50 μg/ml kanamycin and grown overnight to saturation at 37°C. The 2YT media (4 l) supplemented with the same antibiotic concentration were inoculated from the overnight pre-culture and grown at 37°C. When the cell growth reached an OD_600_ of 0.8, expression was induced by adding 0.2 mM IPTG, and incubation continued for another 4 h at 37°C. Cells were harvested by centrifugation at 6000 × *g* for 15 min and resuspended in 5 ml/g of cells in lysis buffer (50 mM Tris–HCl (pH 7.5), 500 mM NaCl, 20 mM imidazole, 10 mM β-mercaptoethanol, 1 mM PMSF, 5% glycerol and one EDTA-free protease inhibitor cocktail tablet per 50 ml) with stirring for 40 min.

Approximately 50–100 ml of the resuspended cells were used for each purification. From here onward, all procedures were performed at 4°C. Cells were sonicated and treated with 3 mg/ml lysozyme (lyophilized protein powder from chicken egg white, purity ≥90%, ≥40 000 units/mg; Sigma-Aldrich, St. Louis, MO, USA) for 1 h to induce cell lysis. The cell debris was removed by centrifugation at 95 834 × *g* for 45 min at 4°C using a Beckman Coulter Optima L-90 K Ultracentrifuge equipped with a 45-Ti rotor. The resulting clear lysate was subjected to several chromatographic steps using the FPLC system. The soluble fractions of the cell lysates for SSB were applied onto a 5 ml HisTrap affinity column pre-equilibrated with buffer A (50 mM Tris–HCl (pH 7.5), 500 mM NaCl, 20 mM imidazole, 10 mM β-mercaptoethanol, and 5% glycerol). The column was washed with 15 column volumes (CV) of buffer A. The protein fusion was eluted with a 15 CV linear gradient of buffer A and 350 mM imidazole. The resulting eluted protein fractions were collected, and His-tagged SUMO protease Ulp1 was added. The resulting mixture was dialyzed against 3 l of buffer A overnight at 4°C. The cleaved protein was then subjected to another 5 ml HisTrap affinity column pre-equilibrated with buffer A, as described above; however, SSB, cleaved from the His-SUMO fusion, was recovered in the flow-through fractions. The flow-through fractions containing cleaved and almost pure SSB were collected, mixed, and concentrated to a 1 ml volume. The concentrated SSB fraction was applied to a 120 ml Superdex 200 pg size-exclusion column pre-equilibrated with 1.5 CV of storage buffer (50 mM Tris–HCl (pH 7.5), 50% glycerol, 300 mM NaCl and 10 mM BME). The pure fractions were selected, mixed, concentrated to 1 ml, aliquoted, flash frozen, and stored at −80°C. All purified proteins are shown in Supplementary Figure S1.

### DNA substrates

The HPLC-purified oligonucleotides used in the ensemble and single-molecule substrate preparation are listed in Supplementary Table S1 (IDT).

*Ensemble L-Tag unwinding substrate*. Forked DNA was formed by annealing the unlabeled leading strand oligo 1 to the Cy5-labelled lagging strand oligo 2 in a 1:1 ratio. NaCl was added to a final concentration of 100 mM, and the mixture was heated to 95°C for 5 min and cooled naturally to room temperature. The annealed product was purified on 10% native PAGE minigels in TBE buffer.

*Ensemble SV40 leading-strand synthesis substrate*. The 3.2-kb primed forked linear DNA was prepared as previously described (49).

*Single-molecule flow-stretching assay substrates*. ssDNA (7.3 knt) and dsDNA (13.5 kb) substrates were prepared as previously described (59–61). The leading strand bead substrate (7 kb) was constructed using a 9-kb bacteriophage λ DNA (New England Biolabs, Ipswich, MA, USA) PCR product with primers 4 and 5, a 6-kb λ DNA PCR product with primers 6 and 7, and a biotinylated oligo 8. An ApaI site was incorporated into the two PCR products using primers 5 and 6. The PCR products were digested with ApaI (New England Biolabs) and ligated with T4 DNA ligase (Invitrogen, Waltham, MA, USA). The ligated product contained two closely located Nt.BsmAI sites, 7 kb away from the 5′ digoxigenin, and the two nicking positions were on the same strand. The ligated product was digested with Nt.BsmAI (New England Biolabs). To create the fork, a 100-fold molar excess of biotinylated oligo 8 was added, and the mixture was heated to 95°C for 5 min and cooled to room temperature naturally. After purification to remove free primers using a QIAGEN PCR purification kit, the substrate was sealed with T4 DNA ligase (Invitrogen). The leading strand bead substrate and its preparation are illustrated in the schematic in Supplementary Figure S2.

### Ensemble L-tag unwinding assays

Reactions contained 25 nM L-Tag and indicated concentration of Mcm10 and/or FPC with 0.5 nM DNA substrate in 20 μl final volume of helicase unwinding buffer (20 mM Tris–HCl (pH 7.5), 5 mM MgCl_2_, 1 mM DTT, 50 mM KCl and 200 μg/ml BSA). Reactions were mixed on ice and incubated for 10 min at 37°C. The indicated concentration of Mcm10 and/or FPC and 4 mM ATP were added. After 1 min of starting the reaction, 25 nM of oligo 3, the unlabeled version of lagging oligo 2, was used as the oligo trap to prevent re-annealing of unwound labeled lagging oligo. After 20 min, reactions were terminated using stop buffer (20 mM EDTA, 0.5% SDS, and 50% glycerol). Reactions were treated with proteinase K and separated on 10% native PAGE minigels in TBE buffer. Gels were scanned using a Typhoon 9400 laser imager (GE Healthcare, Little Chalfont, UK).

### Ensemble SV40 leading-strand synthesis and exchange assays

Reaction systems (10 μl) contained 30 nM Pol δ, 30 nM ΔN-RFC, 100 nM PCNA, 30 nM L-Tag, 100 nM RPA and 1.5 nM linear forked template in replication buffer (25 mM HEPES (pH 7.6), 100 μg/ml BSA, 1 mM DTT, 10 mM Mg acetate, 100 mM K glutamate, 4 mM ATP, 150 μM of each unlabeled dNTP, and 20 μM of labeled dNTP (dCTP)). The leading strand was synthesized by incubating L-Tag with the linear forked DNA for 5 min at 37°C in the presence or absence of the indicated concentration of FPC and Mcm10, followed by the addition of Pol δ, ΔN-RFC, PCNA, RPA, ATP, dNTPs and 10 μCi ^32^P-dCTP. After the indicated reaction time, reactions were quenched using 40 mM EDTA and electrophoresed on 0.7% alkaline agarose gel at 35 V for 17 h. The gel was backed with DE81 paper, compressed, and imaged using Sapphire Biomolecular Imager (Azure Biosystems, Dublin, CA, USA). For the Pol δ exchange experiments, the replisome was assembled as described above, and indicated concentrations of Pol δ polymerase-deficient mutant were added at indicated time point after initiation.

### Single-molecule flow-stretching assays

Flow cell preparation, data acquisition, and analysis were performed as described previously (59,60). All single-molecule flow-stretching assays were performed at 32°C. Briefly, the force extension curves of the two dsDNA substrates were constructed by measuring the length of individual DNA molecules and calculating the drag forces at different flow rates using the equipartition theorem equation (60) and fitting their force extension curves by the Worm-like chain model (59,62,63) (Supplementary Figure S3). The conversion factor from dsDNA to ssDNA at the applied 2.6 pN stretching force (3.76 nt/nm) was extracted from previoulsy published force extension curve for λ DNA (63). Similarly, for the M13 ssDNA substrate, the conversion factor from ssDNA to dsDNA at the applied 2.6 pN stretching force (3.76 nt/nm) was extracted from a previoulsy published force extension curve performed using similar substrate and buffer conditions (64).

To calculate the spatial resolution of the single-molecule assays, baselines of unreplicated DNA molecules were extracted for each substrate and reaction category, and the histograms of baseline bin centers were plotted and fitted with Gaussian distributions. All standard deviations from these histograms were smaller than 200 bp (data not shown). Consequently, the spatial resolution of the single molecule assays was determined as 200 bp.

**Figure 1.**
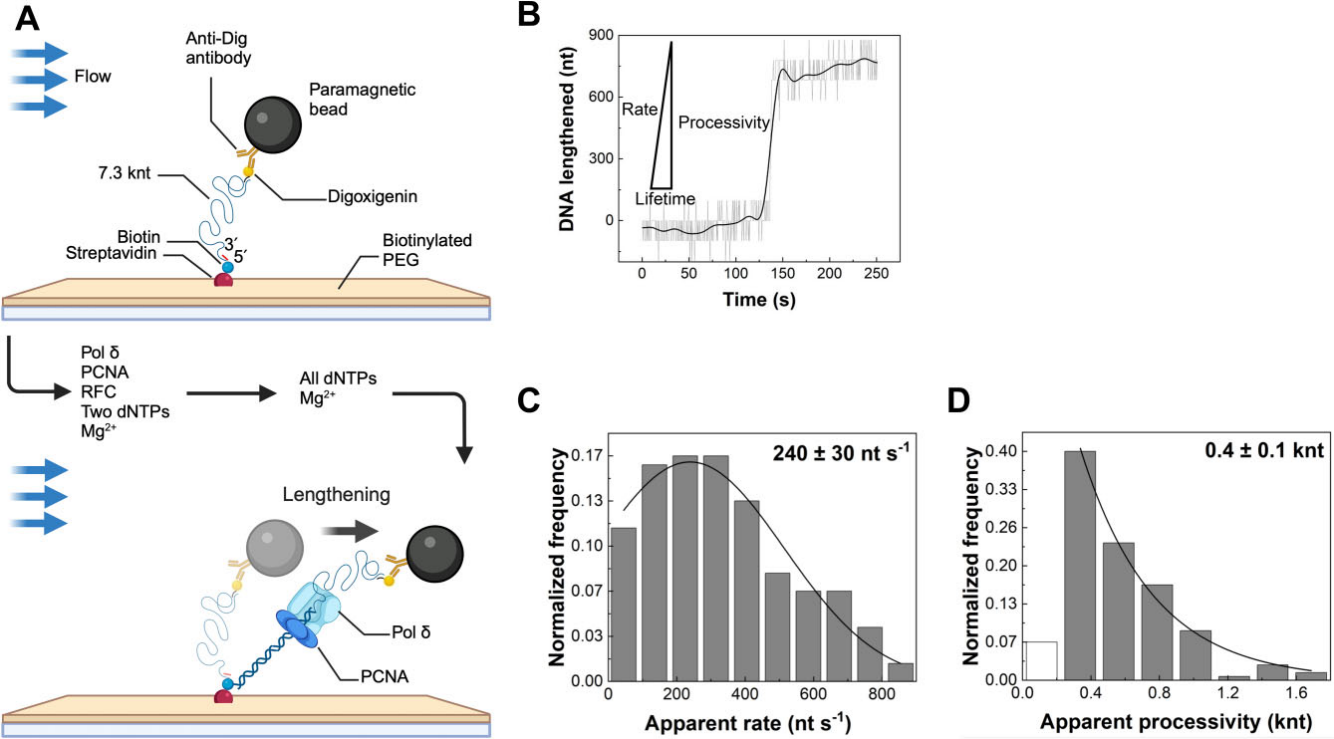
Single-molecule characterization of Pol δ primer extension. (**A**) DNA substrate for primer extension. A primer was annealed to circular M13mp18 ssDNA to introduce Biotin and Digoxigenin at each end separately, with an EcoRI site in the middle. EcoRI restriction enzyme digestion linearized the primer-annealed circular ssDNA to produce a 3′ primer terminus. (**B**) Representative trace showing Pol δ-dependent primer extension. The black line represents smoothing by a Fast Fourier Transform (FFT) filter. One event's rate, processivity, and lifetime are defined as indicated. (**C**) Histogram of Pol δ-dependent primer extension apparent rates. The black line represents a Gaussian fit with a rate of 240 ± 30 nt/s (mean ± SEM) (*n* = 151 events). (**D**) Histogram of Pol δ-dependent primer extension apparent processivities. The black line represents a single exponential decay fit with a processivity of 0.4 ± 0.1 knt (mean ± SEM) (*n* = 151 events). All schematics were created with BioRender.com.

Pauses were initially defined as a minimum of six data points (at acquisition rate of 2 Hz) with amplitude fluctuations less than the standard deviation of the noise. Subsequently, the threshold rate of the pauses was calculated to ensure no DNA synthesis is mistakenly included. At first, the DNA replication and unwinding rates were collected exhaustively. Applying the rule of 3 standard deviations from the mean rates to calculate the threshold rates of pauses would produce negative values. Therefore, the threshold rates of pauses for each category were defined as one tenth of the mean rate, which were 0.1 and 0.5 bp/s for the L-Tag unwinding and SV40 leading-strand synthesis, respectively. Pauses shorter than 3 s, DNA length changes shorter than 200 bp, DNA unwinding events slower than 0.1 bp/s, and DNA replication events slower than 0.5 bp/s would have been buried in the baseline noise if they existed. Therefore, several exponential decay fittings did not include the first bins where these conditions would be categorized (Figure 1D, Figure 3E, Figure 6A, B, Supplementary Figures S17, S20 and S26).

Single-step processivity, single-step lifetime, multi-step processivity, and multi-step lifetime were defined as the following. For one segment of a trace, it is either an event or a pause; they are mutually exclusive. The DNA length change corresponding to an event is named as single-step processivity. When there were more than one event in one trace, separated by pause(s), the total DNA length change was named as multi-step processivity. The single-step processivity is a subset of the multi-step processivity; they are not mutually exclusive. If there was only one event in one trace, the single-step processivity would be equal to the multi-step processivity. Single-step lifetimes and multi-step lifetimes were defined in the same way. It is noteworthy that when there were more than one event on one trace, duration(s) of pause(s) were included in the multi-step lifetimes.

*Primer extension*. First, Pol δ was loaded at the 3′ primer terminus using 40 nM ΔN-RFC, 150 nM PCNA, and 60 nM Pol δ in primer extension buffer (50 mM HEPES–KOH (pH 7.5), 2 mM MgCl_2_, 12 mM Mg acetate, 80 mM KCl, 100 μg/ml BSA, 5 mM DTT, 1 mM ATP and 500 μM dATP) at 32.5 μl/min for 5 min. Reactions were initiated by introducing a primer extension buffer containing no protein or ATP and supplemented with 500 μM dCTP, dGTP and dTTP.

*L-Tag unwinding*. First, L-Tag was loaded onto the fork using 20 nM L-Tag in replication buffer (40 mM Tris–HCl (pH 7.5), 10 mM Mg acetate, 50 mM K glutamate, 100 μg/ml BSA and 1 mM DTT) at 32.5 μl/min for 10 min. Subsequently, the sample was extensively washed by introducing the same replication buffer containing no protein at 32.5 μl/min for 7 min (∼15 chamber volumes). Reactions were initiated by introducing a replication buffer containing no protein (where indicated) or 100 nM RPA (where indicated) or 30 nM Pol δ polymerase-and-exonuclease-deficient mutant (where indicated) and supplemented with 3 mM ATP.

*SV40 leading-strand synthesis*. Pol δ was loaded onto the fork using 30 nM ΔN-RFC, 200 nM PCNA, and 30 nM Pol δ WT (where indicated) or 30 nM Pol δ polymerase-deficient mutant (where indicated) in replication buffer (40 mM Tris–HCl (pH 7.5), 10 mM Mg acetate, 50 mM K glutamate, 100 μg/ml BSA, 1 mM DTT, 3 mM ATP and 100 μM dATP and dGTP) at 32.5 μl/min for 5 min. L-Tag was then loaded onto the fork using 20 nM L-Tag, 16 nM FPC (where indicated), and 16 nM Mcm10 (where indicated) in replication buffer (40 mM Tris–HCl (pH 7.5), 50 mM K glutamate, 100 μg/ml BSA, 1 mM DTT and 100 μM dATP and dGTP) at 32.5 μl/min for 10 min. Subsequently, the sample was extensively washed by introducing the same replication buffer containing no protein at 32.5 μl/min for 7 min (∼15 chamber volumes). Reactions were initiated by introducing 400 nM *E. coli* SSB (where indicated) or 100 nM human RPA (where indicated) or 30 nM Pol δ WT (where indicated) or 30 nM Pol δ polymerase-deficient mutant (as indicated) in replication buffer supplemented with 10 mM Mg acetate, 3 mM ATP and 100 μM dCTP, dGTP, dATP and dTTP.

All representative single-molecule traces with smoothing lines were smoothed by Fast Fourier Transform filters in OriginPro, Version 2024. OriginLab Corporation, Northampton, MA, USA.

### Microscale thermophoresis (MST)

His6-tagged WT Pol δ and Pol δ polymerase-deficient mutants were labeled with RED-Tris-NTA dye in PBS-T buffer (NanoTemper Technology, München, Germany) with a dye-to-protein ratio of 1:2. The reaction mixture was incubated for 30 min at room temperature. The interaction between Pol δ and L-Tag was measured using the MST instrument monolith NT.115 in premium-coated capillaries supplied by NanoTemper Technology. A series of 10 μl reaction mixtures of 16 different L-Tag concentrations were prepared, beginning with 500 nM. Each reaction was mixed with 10 μl of WT Pol δ or Pol δ polymerase-deficient mutant labeled with RED-Tris-NTA to obtain a final concentration of 20 nM. All reactions were performed in PBS-T buffer and incubated for 40 min at 37°C before loading into the capillaries. Fluorescent molecules were excited using a red laser (640 nm) to monitor the spatial distribution of the molecules in each capillary. The thermophoresis signals were measured in each capillary using a temperature gradient induced by an infrared (IR) laser with an emission wavelength of 1480 nm focused on a locally defined sample volume. Upon heating, the protein molecules diffused away from the peak of the temperature, and the bound and unbound proteins responded differently. The change in the thermophoresis of fluorescent molecules due to binding was plotted and used to calculate the bound protein fraction, and the dissociation constant was obtained by fitting the results to a binding isotherm.

## Results

### Primer extension by Pol δ–PCNA

We monitored the rate and processivity of Pol δ–PCNA primer extension at the single-molecule level, as described previously (61), using a well-established flow-stretching bead assay (Figure 1A) (63,65). Briefly, upon primer extension the surface-tethered ssDNA template (short/coiled) was converted to dsDNA (long/stretched), resulting in the overall lengthening of the DNA and movement of the bead in the flow direction (Figure 1A). Pol δ and PCNA were first loaded onto the 3′ primer terminus in a flow buffer containing human RFC, Mg^2+^, ATP and the first two dNTPs to be incorporated. Subsequently, primer extension was initiated using a flowing buffer containing Mg^2+^ and all four dNTPs. Figure 1B shows a typical length change of an individual DNA molecule as a function of time (more examples in Supplementary Figure S4). The rate, processivity, and lifetime of individual DNA molecules were calculated from the slope, DNA length change, and period of DNA length change of the burst phase, respectively (Figure 1B). The applied stretching force in these experiments, ∼2.6 pN, is within the regime forces used in several previous studies on polymerases (61,64–66). It has been suggested that secondary structures may still exist in regime forces below 6 pN (67–70). Therefore, we cannot rule out the possibility that transient pausing below the spatial and temporal resolution of the assay may exist during the burst phase of DNA length change. As a precaution, we will refer to all kinetics derived from the burst change in DNA length throughout the paper as ‘apparent’. The mean rate and processivity were determined by fitting their distribution histograms with Gaussian and single exponential decay functions, respectively, resulting in an apparent rate of 240 ± 30 nt/s (mean ± SEM) (Figure 1C) and an apparent processivity of 0.4 ± 0.1 kb (mean ± SEM) (Figure 1D), consistent with earlier ensemble measurements (71).

### DNA unwinding by L-Tag in the absence and presence of RPA

**Figure 2.**
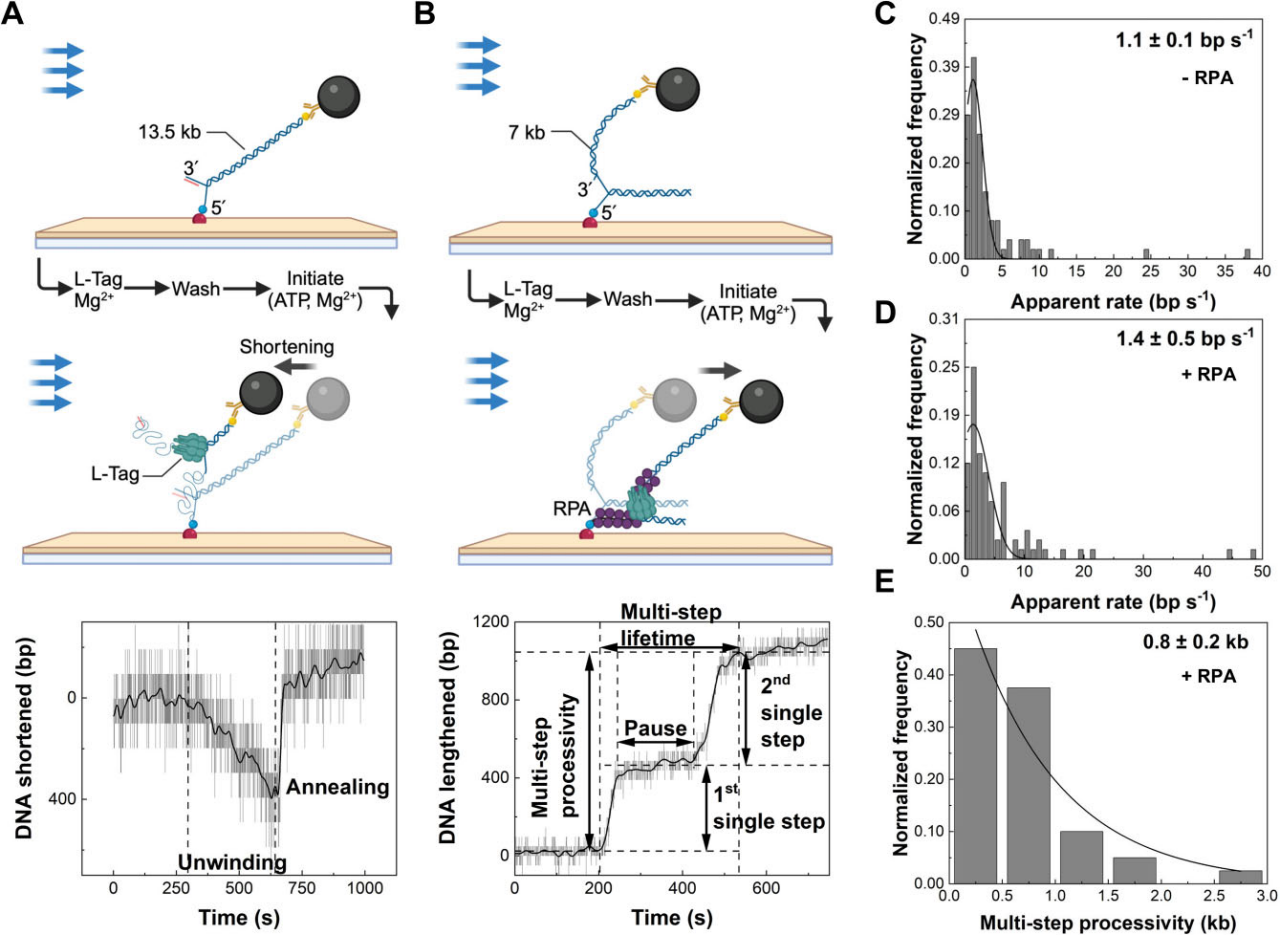
Single-molecule characterization of L-Tag unwinding. (**A**) DNA substrate for L-Tag alone, which contains a replication fork at one end of the 13.5-kb dsDNA fragment and a bead attached at the other end. A representative trace shows L-Tag-dependent dsDNA unwinding. The black line represents smoothing by a FFT filter. (**B**) DNA substrate for L-Tag unwinding with RPA, which contains a replication fork at one end of the 7-kb dsDNA fragment and a bead attached at the same end, at the end of the leading-strand arm of the fork. A representative trace showing L-Tag-dependent dsDNA unwinding with RPA. The black line represents smoothing by a FFT filter. Single-step processivity, pause, multi-step lifetime and multi-step processivity are defined as indicated. (**C**) Histogram of L-Tag-dependent unwinding apparent rates. The black line represents a Gaussian fit with a rate of 1.1 ± 0.1 bp/s (mean ± SEM) (*n* = 51 events). (**D**) Histogram of L-Tag-dependent unwinding apparent rates with RPA. The black line represents a Gaussian fit with a rate of 1.4 ± 0.5 bp/s (mean ± SEM) (*n* = 81 events). (**E**) Histogram of L-Tag-dependent unwinding multi-step processivities with RPA. The black line represents a single exponential decay fit with a processivity of 0.8 ± 0.2 kb (mean ± SEM) (*n* = 40 traces). All schematics were created with BioRender.com.

To visualize helicase unwinding kinetics using L-Tag, a linear dsDNA substrate containing a replication fork was used (Figure 2A). During dsDNA unwinding, the bead movement against the flow direction was observed during the conversion of surface-tethered dsDNA to ssDNA (Figure 2A). A single L-Tag copy was pre-assembled at the fork by flowing L-Tag into a buffer containing Mg^2+^ followed by extensive washing with the same buffer without protein. Unwinding was initiated using a flowing buffer containing Mg^2+^ and ATP. Figure 2A shows the length change of an individual DNA molecule as a function of time (more examples in Supplementary Figure S5A). Gradual shortening of the DNA indicates the sustained conversion of dsDNA into ssDNA, followed by the re-annealing of unwound ssDNA strands. The events were characteristically short and statistically unreliable in determining processivity because fitting their distribution resulted in a value below the 0.2 kb spatial resolution of our assay (Supplementary Figure S5B). Nonetheless, their slopes were still reliably fit to obtain an apparent dsDNA unwinding rate of 1.1 ± 0.1 bp/s (mean ± SEM) (Figure 2C), consistent with earlier single-molecule and ensemble measurements (36,37,72).

Next, we investigated the effect of coating unwound ssDNA strands with *E. coli* SSB or human RPA on L-Tag unwinding activity. We first examined the effect of SSB protein binding on the ssDNA contour length at saturating protein concentrations. *E. coli* SSB increased the contour length by 26 ± 1%, whereas RPA increased it to the same length as dsDNA (98 ± 1%) (Supplementary Figure S6), which is consistent with the results of a previous study (12). Therefore, RPA is unsuitable for visualizing the changes in DNA length during L-Tag unwinding using the substrate configuration shown in Figure 2A. To overcome this, we added 7 kb dsDNA to the leading-strand arm at the fork and attached the bead to its end; the 7 kb arm was used to keep the bead away from the surface (Figure 2B). In this configuration, during DNA unwinding, the bead movement along the flow direction indicates the elongation of the RPA-coated ssDNA on both the leading and lagging strands (Figure 2B). Figure 2B shows the length change of an individual DNA molecule as a function of time (more examples in Supplementary Figure S7). In contrast to the DNA re-annealing in Figure 2A and Supplementary Figure S5A, a pause appears at the end of the unwinding event. Restarting dsDNA unwinding after pausing was also observed in a few traces (Figure 2B and Supplementary Figure S7), where each step of DNA unwinding seprated by >3 s pausing is referred to as a single step, and the accumulative single steps with pausing are referred to as a multistep (Figure 2B).

The apparent rate of dsDNA unwinding derived from the single steps was 1.4 ± 0.5 bp/s (mean ± SEM) (Figure 2D), and the accumulative processivity derived from the multisteps was 0.8 ± 0.2 kb (mean ± SEM) (Figure 2E). These results demonstrate that RPA does not change the L-Tag unwinding rate but increases its processivity significantly.

### Leading-strand synthesis by L-Tag, Pol δ–PCNA and RPA

**Figure 3.**
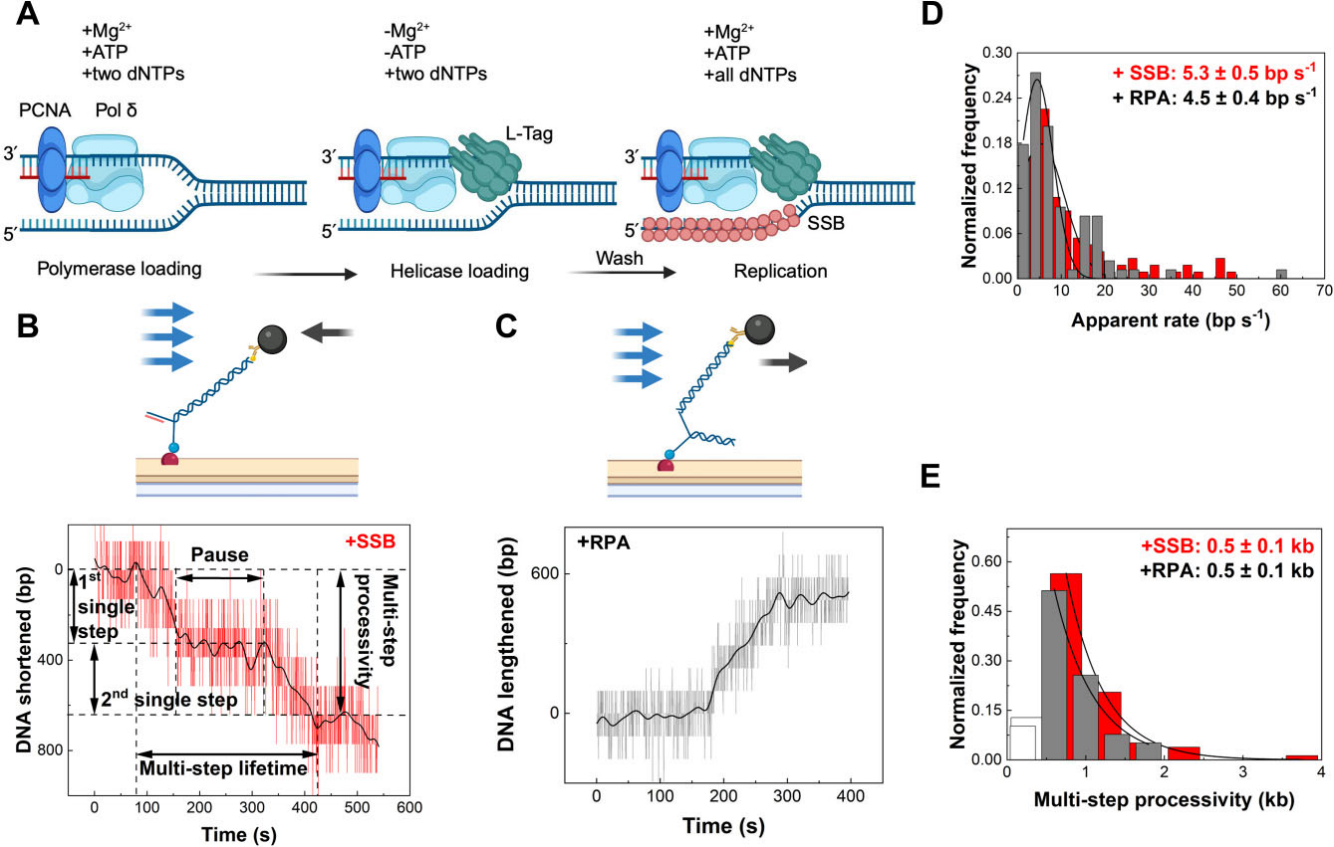
Single-molecule characterization of SV40 leading-strand synthesis. (**A**) SV40 replisome pre-assembly steps. DNA substrates and beads were tethered in the flow cell, and Pol δ and PCNA were loaded with RFC (not illustrated) at the replication fork. The two dNTPs were added to prevent the exonuclease activity of Pol δ, and L-Tag was loaded. The two dNTPs were still present to prevent Pol δ exonuclease activity. Mg^2+^ and ATP were removed to prevent L-Tag helicase activity. The following extensive wash retained the same buffer conditions but contained no protein. To begin SV40 leading-strand synthesis, Mg^2+^, ATP and all four dNTPs are added, including single-strand-binding proteins. (**B**) The simplified schematic of the DNA substrate in Figure 2A. The representative trace in red shows SV40 replisome-dependent leading-strand synthesis with *E. coli* SSB compatible with it. The black line represents smoothing by a FFT filter. Single-step processivity, pause, multi-step lifetime and multi-step processivity are defined as indicated. (**C**) The simplified schematic of the DNA substrate in Figure 2B. The representative trace in grey shows SV40 replisome-dependent leading-strand synthesis with RPA compatible with it. The black line represents smoothing by a FFT filter. (**D**) Histograms of SV40 replisome-dependent leading-strand synthesis apparent rates. The one with RPA is in grey, fitted by Gaussian distribution with a rate of 4.5 ± 0.4 bp/s (mean ± SEM) (*n* = 84 events). The one with *E. coli* SSB is in red, fitted by Gaussian distribution with a rate of 5.3 ± 0.5 bp/s (mean ± SEM) (*n* = 111 events). (**E**) Histograms of SV40 replisome-dependent leading-strand synthesis multi-step processivities. The one with RPA is in grey, fitted by single exponential decay distribution with a processivity of 0.5 ± 0.1 kb (mean ± SEM) (*n* = 39 traces). The one with *E. coli* SSB is in red, fitted by single exponential decay distribution with a processivity of 0.5 ± 0.1 kb (mean ± SEM) (*n* = 78 traces). All schematics were created with BioRender.com.

We monitored leading-strand synthesis in the presence of *E. coli* SSB using the substrate shown in Figure 2A or RPA using the substrate in Figure 2B. To pre-assemble a single copy of the L-Tag–Pol δ–PCNA complex, we loaded Pol δ and PCNA onto the fork in a flowing buffer containing RFC, Mg^2+^, ATP and the first two dNTPs to be incorporated. Subsequently, the L-Tag was loaded onto the fork in a flowing buffer containing ATP and the two dNTPs, and excess L-Tag was removed by extensive washing using the same buffer without protein. Finally, replication was initiated by adding a flowing buffer containing Mg^2+^, ATP, all four dNTPs, and SSB protein (Figure 3A). Similar to DNA unwinding by L-Tag, the leading-strand DNA synthesis time traces display either single-step or multi-step events (Figure 3B, C, Supplementary Figures S8 and S9). The apparent rate of leading-strand synthesis derived from single steps with *E. coli* SSB was 5.3 ± 0.5 bp/s (mean ± SEM) (Figures 3D and Supplementary Figure S8) and with RPA 4.5 ± 0.4 bp/s (mean ± SEM) (Figure 3D and Supplementary Figure S9). These rates differed significantly from those of Pol δ–PCNA primer extension or L-Tag unwinding, demonstrating that the observed bead movements were replisome-dependent. Interestingly, similar to DNA unwinding by L-Tag, the rate of leading-strand synthesis was unaffected by *E. coli* SSB or RPA (Supplementary Figure S10), demonstrating that L-Tag acceleration during leading-strand synthesis was due to the polymerase. However, similar to DNA unwinding by the L-Tag, the apparent single-step processivity events of leading-strand synthesis were short, and fitting their distribution resulted in a value of 0.3 ± 0.1 kb (mean ± SEM) (Figure 5D), which is at the borderline of the 0.2 kb spatial resolution of the assay. Consequently, the multi-step processivity in the presence of *E. coli* SSB and RPA was 0.5 ± 0.1 kb (mean ± SEM) in each case (Figure 3E). The apparent pause duration for the single replisomes that restarted DNA synthesis was 182 ± 23 s (mean ± SEM) (Figure 5D, Supplementary Figures S20C and S23). This pausing and restarting behavior from single copies of the leading-strand complex is consistent with previous findings, suggesting that the replisome naturally pauses during synthesis (73,74). The similar kinetics of DNA unwinding and leading-strand synthesis in the substrates shown in Figure 2A and B indicate that the different force pulling geometries relative to the helicase doesn’t affect the results under our regime force. In fact, the force required to open a DNA hairpin is >16 pN (75), which is much higher than the ∼3 pN force regime applied in this study. In addition, several single-molecule magnetic tweezers experiments show that L-Tag unwinds the dsDNA at rates similar to those reported in our study under different regime forces (36,37).

For subsequent experiments, leading-strand synthesis was performed in the presence of *E. coli* SSB because the bead movement against the flow produces less noise than when the bead moved in the flow direction.

### Human FPC and Mcm10 are factors of the SV40 replisome

**Figure 4.**
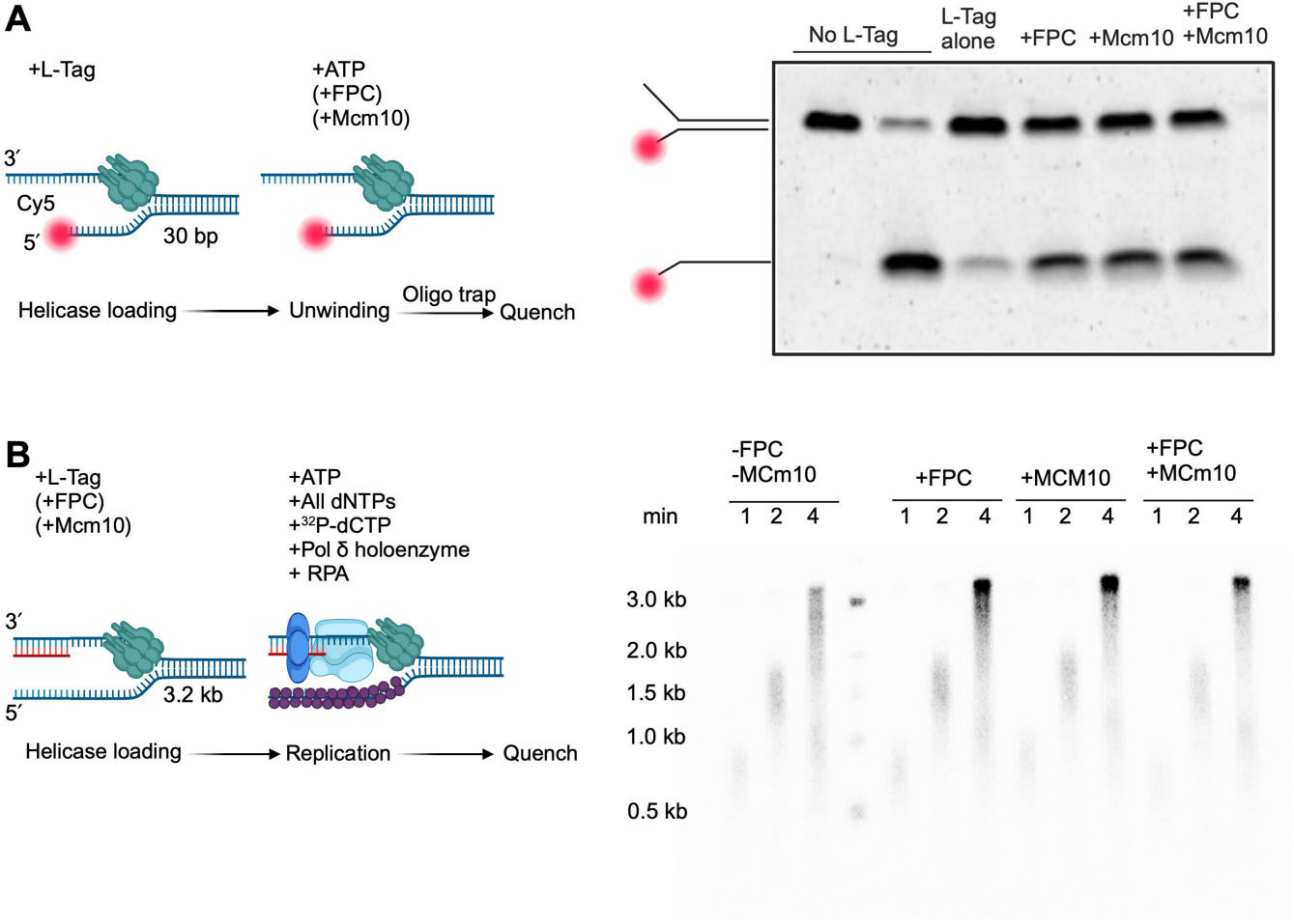
FPC and Mcm10 effects in bulk. (**A**) Schematic of L-Tag unwinding bulk assay and native PAGE gel of L-Tag unwinding products, 5′ Cy5-labelled. The DNA substrate was 0.5 nM. L-Tag was 25 nM. FPC and Mcm10 were 15 nM when indicated. Both FPC and Mcm10 stimulated L-Tag unwinding. (**B**) Schematic of SV40 leading-strand synthesis bulk assay and alkaline agarose gel of SV40 leading-strand synthesis products. Schematic of the substrate and reaction steps (Left). Product of DNA replication visualized via ^32^P-dCTP-labelled (nucleotide incorporation). The DNA substrate was 1.5 nM. L-Tag was 30 nM. FPC and Mcm10 were 15 nM when indicated. All schematics and gel labelling were created with BioRender.com.

The establishment of reconstituted yeast (49) and human replisomes (17) provided an opportunity to revisit the components of the SV40 replisome beyond the historic minimal replisome. We focused on FPC and Mcm10, which interact with the human CMG complex (16). Previous study showed that FPC co-immunoprecipitates with L-Tag, indicating an interaction between the two proteins (54). We first conducted bulk-phase DNA unwinding assays using L-Tag in the presence of FPC and/or Mcm10 (Figure 4A). Interestingly, the L-Tag unwinding efficiency increased in the presence of FPC alone, Mcm10 alone, and FPC and Mcm10 (Figure 4A). The saturation concentrations of FPC and Mcm10 did not exceed 8 nM, although the L-Tag concentration was 25 nM (Supplementary Figure S11). However, the template concentration was limited, suggesting that Mcm10 and FPC may preferentially interact with L-Tag at the fork.

Next, we investigated the effects of FPC and Mcm10 on SV40 leading-strand synthesis in bulk using a 3.2-kb forked substrate. L-Tag was pre-assembled at the fork in the presence of FPC and/or Mcm10 in the presence of Mg^2+^. The reaction was initiated by adding Pol δ, RFC, PCNA, ATP, Mg^2+^, and dNTPs (Figure 4B). Time point- and concentration-dependent replication assays showed that both FPC and Mcm10 stimulated leading-strand synthesis (Figure 4B and Supplementary Figure S12). Besides, stimulation by FPC and Mcm10 saturated at lower concentrations relative to L-Tag was observed, consistent with the observation for stimulation of L-Tag unwinding alone (Supplementary Figure S12).

### FPC and Mcm10 attenuates restarting, processivity, and rate of leading-strand synthesis

**Figure 5.**
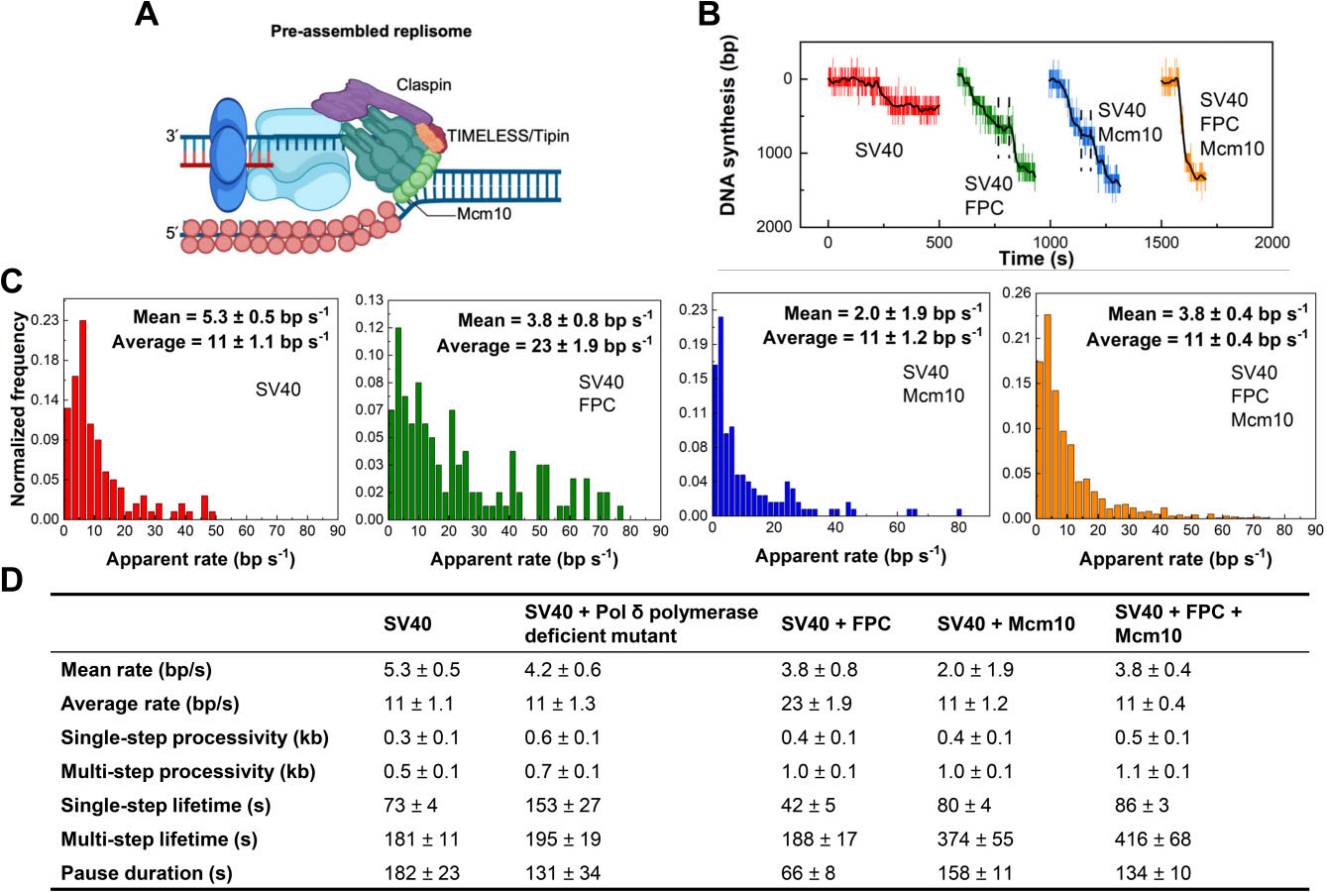
FPC and Mcm10 effects on SV40 leading-strand synthesis in single-molecule. (**A**) Schematic of SV40 leading-strand synthesis with human FPC and Mcm10. (**B**) Representative traces showing SV40 leading-strand synthesis (red), SV40 leading-strand synthesis with FPC (olive), SV40 leading-strand synthesis with Mcm10 (blue), and SV40 leading-strand synthesis with FPC and Mcm10 (orange). Black lines represent smoothing by FFT filters. Dashed lines indicate pauses. (**C**) Histograms of SV40 leading-strand synthesis apparent rates. For SV40 alone, the Gaussian fit gives a mean rate of 5.3 ± 0.5 bp/s (mean ± SEM). The arithmetic average rate is 11 ± 1.1 bp/s (average ± SE) (n = 111 events). For SV40 with FPC, the Gaussian fit gives a mean rate of 3.8 ± 0.8 bp/s (mean ± SEM). The arithmetic average rate is 23 ± 1.9 bp/s (average ± SE) (*n* = 121 events). For SV40 with Mcm10, the Gaussian fit gives a mean rate of 2.0 ± 1.9 bp/s (mean ± SEM). The arithmetic average rate is 11 ± 1.2 bp/s (average ± SE) (*n* = 131 events). For SV40 with FPC and Mcm10, the Gaussian fit gives a mean rate of 3.8 ± 0.4 bp/s (mean ± SEM). The arithmetic average rate is 11 ± 0.4 bp/s (average ± SE) (*n* = 973 events). (**D**) All statistics of SV40 leading-strand synthesis. Their actual distributions are presented in Supplementary Figures S16, S18, S19, S21–S23. All schematics were created with BioRender.com.

To clarify the functions of the SV40 replisome-associated FPC and Mcm10, we performed single-molecule flow-stretching bead assays. Single copies of replisome-associated FPC, Mcm10 or both FPC and Mcm10 were obtained by loading them with L-Tag and performing extensive washing with buffer without proteins (Figure 5A). Figure 5B shows the representative replication events for each combination, and more examples are shown in Supplementary Figures S13–S15. The rate distribution of FPC-mediated leading-strand synthesis showed an increase in the faster population spread across the long tail. The rate distributions for Mcm10-mediated leading-strand synthesis resembled those of the SV40 replisome alone, and adding Mcm10 reverted the SV40 and FPC mixed rate distributions to those of the SV40 replisome alone (Figure 5C and Supplementary Figure S16).

To better understand the differences between these replisome assemblies, we characterized the single-step and multi-step kinetics and pause duration before replication restart (Figure 5D, Supplementary Figures S17–S23). Compared to the SV40 replisome alone, all apparent single-step processivities moderately increased in the presence of FPC, Mcm10, or both Mcm10 and FPC (Supplementary Figures S17A and S18). However, the apparent single-step lifetime was only changed when adding FPC, reduced by nearly 50% compared to the SV40 replisome alone, validating that FPC stimulates the rate of DNA synthesis (Supplementary Figures S20A and S21). Interestingly, the number of restarts increased with the addition of FPC and Mcm10 (Supplementary Figure S24B), resulting in a 2-fold increase in the multi-step processivity (Figure 5D, Supplementary Figures S17B, and S19). Further analysis of the apparent pause duration before restarting showed that only FPC significantly shortened the apparent pause duration, demonstrating that FPC may increase the intrinsic probability of restarting DNA synthesis (Supplementary Figures S20C and S23). The apparent pause duration with the Mcm10 addition was similar to that of SV40 alone (Supplementary Figures S20C and S23). The increased restart population with the Mcm10 addition resulted in a nearly 2-fold increase in the multi-step lifetime of the SV40 replisome (Supplementary Figures S20B and S22), which represents the total time the replisome spent on the DNA including the apparent pause durations (Figure 3B), demonstrating that Mcm10 is critical for stabilizing the replisome at the fork. In contrast, a faster apparent rate and reduced apparent pause duration with the FPC addition resulted in a multi-step lifetime similar to that of SV40 alone (Supplementary Figures S20B and S22). The addition of both Mcm10 and FPC reverted the apparent pause duration, multi-step processivity, and multi-step lifetime to those of SV40 with Mcm10 (Figure 5D), rendering it challenging to ascertain whether FPC and Mcm10 can coexist at the replication fork. In a control experiment, we skipped the extensive wash step and showed that the apparent rate distributions, apparent pause durations, and all apparent single-step and multi-step statistics were highly consistent with those with the extensive wash step (data not shown). This confirmed that FPC and Mcm10 form functionally stable complexes at the SV40 fork. Consistent with this conclusion, transition plots of the rates with DNA synthesis restarting showed that the distances from the diagonal lines were similar regardless of the presence of FPC and Mcm10 (Supplementary Figure S24A), indicating that FPC and Mcm10 do not form subpopulations of complexes with different kinetics.

### Pol δ cannot be exchanged during the intrinsic processivity of SV40 leading-strand synthesis

**Figure 6.**
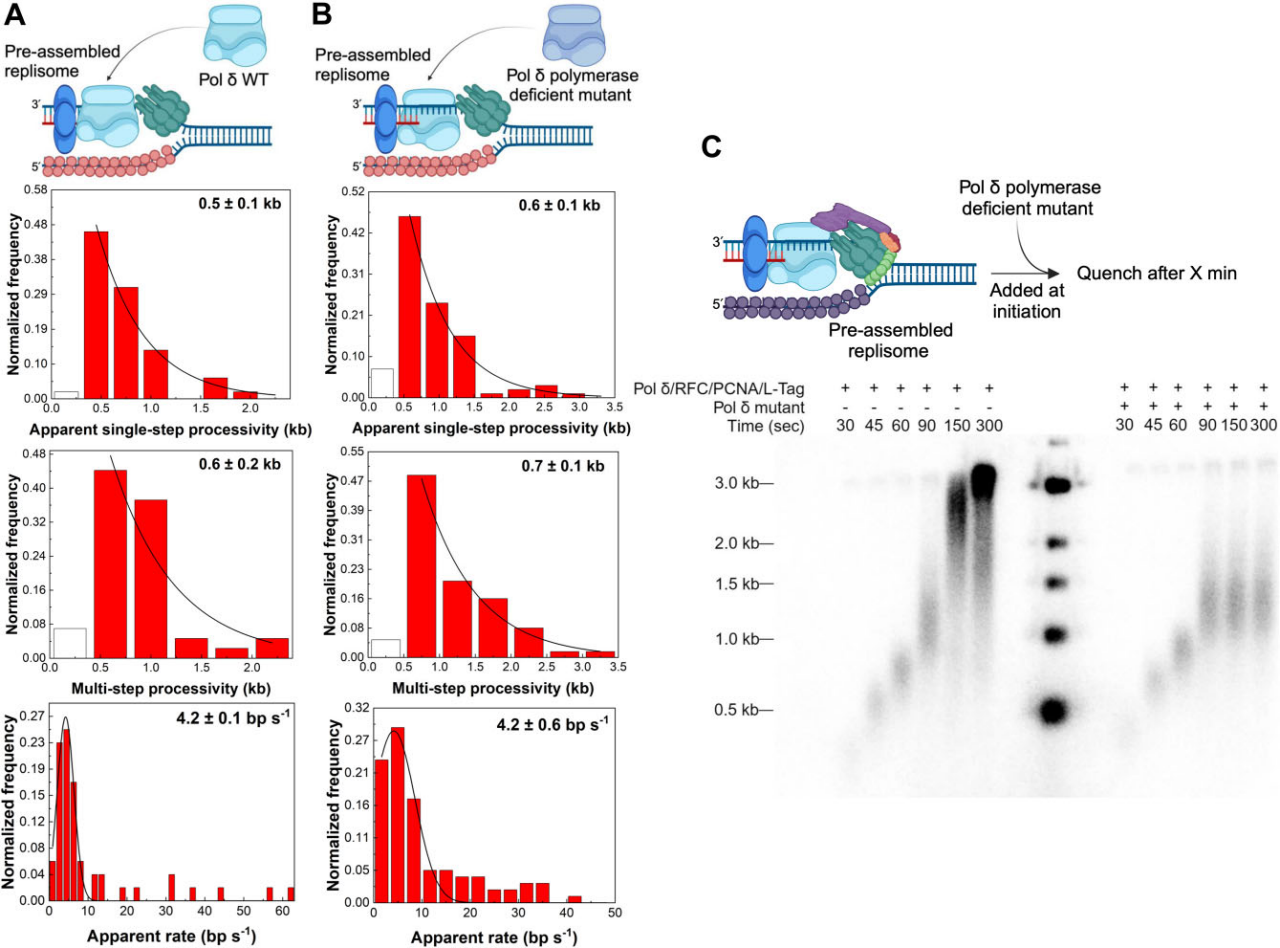
Polymerase exchange assays. (**A**) Schematic: SV40 leading-strand synthesis with polymerase exchange. As DNA replication starts, Pol δ WT is present together with *E. coli* SSB. Top panel: histogram of SV40 leading-strand synthesis apparent single-step processivities. The black line represents a single exponential decay fit with a processivity of 0.5 ± 0.1 kb (mean ± SEM) (n = 52 traces). Middle panel: histogram of SV40 leading-strand synthesis multi-step processivities. The black line represents a single exponential decay fit with a processivity of 0.6 ± 0.2 kb (mean ± SEM) (n = 43 traces). Lower panel: histogram of SV40 leading-strand synthesis apparent rates has no difference from the normal one. The black line represents a Gaussian fit with a rate of 4.2 ± 0.1 bp/s (mean ± SEM) (*n* = 52 events). (**B**) Schematic: SV40 leading-strand synthesis with polymerase exchange. As DNA replication starts, Pol δ polymerase-deficient mutant is present together with *E. coli* SSB. Top panel: histogram of SV40 leading-strand synthesis apparent single-step processivities. The black line represents a single exponential decay fit with a processivity of 0.6 ± 0.1 kb (mean ± SEM) (*n* = 96 traces). Middle panel: histogram of SV40 leading-strand synthesis multi-step processivities. The black line represents a single exponential decay fit with a processivity of 0.7 ± 0.1 kb (mean ± SEM) (*n* = 64 traces). Lower panel: histogram of SV40 leading-strand synthesis apparent rates has no difference from the normal one. The black line represents a Gaussian fit with a rate of 4.2 ± 0.6 bp/s (mean ± SEM) (*n* = 94 events). (**C**) Pol δ polymerase-deficient mutant terminates SV40 leading-strand synthesis after certain processivity, time titration. Schematic of Pol δ polymerase-deficient mutant exchange bulk assays and alkaline agarose gels of SV40 leading-strand synthesis products, ^32^P-dCTP-labelled (nucleotide incorporation). Reactions were performed as described in Materials and methods. All schematics and gel labelling were created with BioRender.com.

The increased lifetime of Pol δ–PCNA on DNA from 2 s to 3 min in the presence of L-Tag (Figure 5D, Supplementary Figures S20B and S22) indicates the formation of a complex between Pol δ and L-Tag, which was confirmed using MST binding assays, showing that Pol δ and L-Tag formed a complex with a dissociation binding constant of 6.7 ± 2 nM (mean ± SEM) (Supplementary Figure S25A). The yeast replisome exchanges its replicative polymerases with free polymerases from the solution without altering the rate of DNA synthesis but with slow kinetics of one polymerase/10 min (48), suggesting that the polymerase may fall off the DNA but is retained within the replisome to ensure quick rebinding. To test whether a similar mechanism exists in the SV40 replisome, we pre-assembled a single copy of the leading-strand complex with WT Pol δ and added an excess amount of either WT Pol δ or Pol δ polymerase-deficient mutant for DNA polymerization (Figure 6A, B).

Remarkably, the apparent single-step processivity of leading-strand synthesis in the presence of an excess amount of WT Pol δ increased from 0.3 to 0.5 ± 0.1 kb (mean ± SEM) (Figure 6A and Supplementary Figure S17A), close to its multi-step processivity of 0.6 ± 0.2 kb (mean ± SEM) (Figure 6A). This increase could be due to the restarting of DNA synthesis, in which the pause duration before restarting was below the temporal resolution of our assay. However, the apparent single-step (0.6 ± 0.1 kb (mean ± SEM); Figure 6B) and multi-step (0.7 ± 0.1 kb (mean ± SEM); Figure 6B) processivities increased to a similar extent when excess Pol δ polymerase-deficient mutant was used. Additionally, the apparent rate of leading-strand synthesis was similar in the presence of excess amount of WT Pol δ compared to that of Pol δ polymerase-deficient mutant, whereas the apparent single-step lifetime increased to the same level as the multi-step lifetime (Figure 6A, B and Supplementary Figure S25C, D). In a control experiment, we showed that the addition of Pol δ polymerase-and-exonuclease-deficient mutant did not change the L-Tag apparent unwinding rate, but it somewhat enhanced the apparent unwinding processivity beyond the 0.2 kb spatial resolution of the assay (Supplementary Figure S26), similar to that observed in the SV40 replisome case (Figure 5D and Supplementary Figure S17A). Collectively, these results suggest that the increase in the intrinsic processivity of the pre-assembled leading-strand complex in the presence of excess polymerases is likely due to L-Tag stabilization when interacting with more than one Pol δ.

The inability of the Pol δ polymerase-deficient mutant to reduce the apparent single-step processivity also demonstrates that excess Pol δ in solution cannot be exchanged with the replisome-associated Pol δ during the intrinsic processivity regime of leading-strand synthesis. In a control experiment, we reversed the scheme by pre-assembling a leading-strand complex containing the Pol δ polymerase-deficient mutant, challenged it with WT Pol δ, and observed no DNA synthesis (data not shown). Furthermore, the pre-assembled single replisome restarting of DNA synthesis after an apparent pause duration of 131 ± 34 s (mean ± SEM) (Supplementary Figure S25B) in the presence of an excess amount of Pol δ polymerase-deficient mutant indicates that the replicating polymerase is bound to the primer–template during these long pauses, highlighting the high stability of the leading-strand complex at the replication fork.

We next performed the polymerase exchange experiment using bulk-phase replication assays in the presence of FPC and Mcm10, where we preassembled the repilosme with WT Pol δ and started the reaction in the presence of equimolar concentration of Pol δ polymerase-deficient mutant (Figure 6C). The results showed that leading-strand synthesis progressed similarly to the reaction that did not include the addition of Pol δ polymerase-deficient mutant for the first 90 s before the reaction stopped (Figure 6C). The length of the product upon polymerase exchange was ∼1.1 kb, consistent with the 1.1 kb multi-step processivity of the leading-strand complex in the presence of FPC and Mcm10 from the single-molecule measurement (Figure 5D). These results indicate that leading-strand synthesis progressed until the replisome-associated WT Pol δ dissociated from DNA within its intrinsic processivity regime and was exchanged by the Pol δ polymerase-deficient mutant. In control experiments, we showed that the used concentration of the Pol δ polymerase-deficient mutant was saturating to compete with the dissociated WT Pol δ (Supplementary Figure S27) and similar results were obtained when the exchange experiment was performed in the absence of FPC and Mcm10 (Supplementary Figure S28).

Collectively, the single-molecule and the bulk-phase exchange experiments support the conclusion that Pol δ does not exchange within the intrinisic processivity regime of leading-strand synthesis and that exchange happened as a consequence of Pol δ dissociation from L-Tag. This intrinisic regime of exchange in SV40 was much shorter than that of Pol ϵ in yeast (48), indicating that Pol ϵ forms a more stable leading-strand complex with CMG than Pol δ with L-Tag, consistent with the higher processivity of yeast leading-strand synthesis compare to that of SV40 (12).

## Discussion

This study systematically reconstituted SV40 leading-strand synthesis kinetics at the single-molecule level using several purified proteins. The reported kinetics of primer extension by Pol δ–PCNA, dsDNA unwinding by L-Tag and its influence by RPA, leading-strand synthesis by Pol δ–PCNA, L-Tag and RPA, the stability of Pol δ–PCNA alone and when coupled with L-Tag, and the newly established factors Mcm10 and FPC have significantly advanced our knowledge of this eukaryotic model replication system. The overall kinetics of leading-strand synthesis resembled those of the yeast replisome characterized using similar single-molecule flow-stretching assays (12), highlighting a high degree of conservation among eukaryotic replisomes. However, the underlying kinetics of the partial activities of SV40 displayed some differences compared to those of yeast.

A comparison of primer extensions between human and yeast Pol δ–PCNA showed that their rates of DNA synthesis were similar, approaching 240 nt/s (Figure 1C) (76,77). However, the processivity of the human Pol δ–PCNA was only 0.4 knt (Figure 1D), whereas that of yeast reached several knt (8,78–80). The structures of human and yeast Pol δ–PCNA bound to DNA showed that although the two polymerases displayed similar structures, the yeast PCNA formed 50% more interactions with Pol δ compared to human Pol δ–PCNA (57,81). The shorter lifetime of the human Pol δ–PCNA on DNA suggests that extrapolating information on Pol δ function from yeast to humans is not straightforward. For example, during the maturation of Okazaki fragments, Pol δ–PCNA mediates limited strand displacement synthesis upon encountering the previous Okazaki fragments to displace the primer as a 5′ flap, which is then cleaved by a PCNA-bound Flap Endonuclease 1 (FEN1). In yeast, highly processive 1-nt iterative cycles between PCNA-bound Pol δ and FEN1 remove primers (80). In contrast, in humans, the 1-nt iterative cycles are ∼50-fold slower due to the instability of Pol δ on PCNA, compromising its ability to release FEN1 during the iterative cycles (82). Interestingly, the lifetime of human Pol δ–PCNA is similar to those of bacteriophage T7 DNA polymerase and *E. coli* Pol III holoenzyme (83). Therefore, yeast may characteristically develop a much more stable Pol δ–PCNA complex.

When Pol δ–PCNA was coupled with an L-Tag during leading-strand synthesis, its lifetime increased from 2 s to 3 min (Supplementary Figure S20B), likely due to the low nanomolar dissociation binding constant for the Pol δ–PCNA and L-Tag complex (Supplementary Figure S25A). Additionally, this stability was confirmed in a polymerase exchange experiment, where excess WT Pol δ in the solution failed to rescue pre-assembled leading-strand complexes containing Pol δ polymerase-deficient mutant (data not shown), and vice versa. These results highlight that in single-molecule SV40 leading-strand synthesis, all events are SV40 replisome-dependent, rather than uncoupled DNA unwinding by the L-Tag, followed by gap closure by Pol δ–PCNA. Interestingly, the excess Pol δ polymerase-deficient mutant in the solution enhanced the processivity of the pre-assembled leading-strand replisome with WT Pol δ (Figure 6B). The excess Pol δ polymerase-deficient mutant is anticipated to interact with and stabilize L-Tag, indicating that the L-Tag can recruit extra Pol δ to support lagging-strand synthesis. A comparison of SV40 leading-strand synthesis by L-Tag, Pol δ–PCNA and *E. coli* SSB with yeast CMG, Pol ϵ and *E. coli* SSB using similar single-molecule flow-stretching assays demonstrated that both had comparable rates of 4–5 bp/s; however, yeast leading-strand synthesis was 2-fold more processive (Figure 3E) (12). This demonstrates that the kinetics of leading-strand synthesis are conserved between the two systems, regardless of the replicative polymerase category and the significantly lower stability of the human Pol δ–PCNA complex, underscoring the role of the helicase in stabilizing the leading-strand polymerase. However, polymerase exchange experiments illustrate that Pol δ dissociates from the replisome and is exchanged within 1.5 min in SV40, while in yeast Pol ϵ requires ∼10 min to be exchanged (48), suggesting that Pol ϵ forms a more functionally stable binding with the CMG than Pol δ with L-Tag during leading-strand synthesis. It also suggests that Pol δ forms less dynamic interactions with L-Tag compared to Pol ϵ with CMG.

L-Tag alone unwinds DNA at approximately 1 bp/s (Figure 2C), consistent with previous results (36,37,72), demonstrating 2–10 folds faster rate than that with CMG alone (37). However, L-Tag processivity was below the 0.2 kb detection limit of our single-molecule flow-stretching assay, whereas CMG had a processivity of ∼0.8 kb (37), demonstrating that CMG forms a more stable complex at the fork. RPA increased L-Tag processivity to ∼0.8 kb without altering its rate (Figure 2D, E) while enhancing the CMG rate to ∼5-fold faster than the L-Tag rate (46). This difference in modulation by RPA can be explained by differences in the CMG and L-Tag engagement model with dsDNA (46). L-Tag also showed similarities with CMG concerning using FPC and Mcm10 but displayed a more functionally stable binding to these proteins than CMG. A single copy of the SV40 replisome containing Mcm10 and/or FPC can be pre-assembled, in contrast to the yeast leading-strand replisome, in which MTC may form a transient complex with CMG (12). The observation that L-Tag complexes with FPC and Mcm10 at the fork were saturated at limiting concentrations of FPC and Mcm10 suggests that these complexes are likely mediated by protein–protein interactions and the fork (Supplementary Figures S11 and S12). This is consistent with the structures of the human CMG with FPC and yeast CMG with MTC and Mcm10, showing that these proteins form complexes with CMG ahead of the helicase and interact with the fork (14,16). In these cryo-EM structures, yeast Mrc1 (14) and human Claspin (16) were unstructured, which may explain why MTC transiently interacts with the yeast leading-strand synthesis complex *in vitro* (12).

By comparing the rate, processivity, lifetime, and restart of a single copy of the SV40 leading-strand replisome in the presence of FPC and/or Mcm10 (Figure 5D), we provided a detailed mechanistic understanding of their contribution to the kinetics of leading-strand synthesis. A common feature in their kinetics is the enhancement of the restarting DNA synthesis percentage, resulting in a ∼2-fold enhancement in the multi-step processivity of leading-strand synthesis. The kinetics of the single step showed that FPC significantly increased the rate of leading-strand synthesis by 2-fold but reduced its lifetime, whereas Mcm10 slightly affected its rate and lifetime. The multi-step lifetime increased by 2-fold in the case of Mcm10, demonstrating that it contributes to the replisome stability. These results are consistent with the effect of Mcm10 and MTC on yeast leading-strand synthesis, in which MTC enhances its rate, and both MTC and Mcm10 increase the probability of restarting DNA synthesis and multi-step processivity (12). Interestingly, the pause duration before restarting DNA synthesis was significantly reduced in the presence of FPC, demonstrating that FPC stabilizes the replisome by reducing its pausing duration. In yeast, the C-terminus of Mrc1 might interact with Pol ϵ non-catalytic domain around Cdc45 (14). Considering the similarity of the spatial arrangements between Claspin in the human replisome and Mrc1 in the yeast replisome, the structure of human CMG bound to FPC suggests that the Claspin subunit interacts with Pol ϵ and CMG (16) and may provide an anchor for Pol ϵ at the SV40 replisome. The increase in the Mcm10-induced restart probability without affecting the pause duration suggests that Mcm10 functions as an activator of the CMG complex during DNA replication by stabilizing CMG or overcoming possible obstacles. Furthermore, Mcm10 activates Mcm2–7 during replication initiation (20,84), suggesting that Mcm10 helps activate and stabilize the L-Tag in our *in vitro* leading-strand system through a similar mechanism. Additionally, Mcm10 may stabilize Pol δ or enhance its synthesis activity as Mcm10 interacts with PCNA (85). However, whether FPC and Mcm10 can simultaneously bind to L-Tag remains unclear from our findings. Nonetheless, if such a complex is formed, Mcm10 appears to play a more dominant role because the kinetics of leading-strand synthesis in the presence of both FPC and Mcm10 was closer to that of Mcm10 alone.

Our results provide a comprehensive analysis of the modulation of L-Tag processivity and rate by different proteins. RPA significantly enhanced L-Tag processivity without altering its rate, whereas Pol δ–PCNA reduced its processivity but enhanced its rate in the SV40 leading-strand complex. Excess Pol δ enhanced the processivity of the leading-strand complex, suggesting that L-Tag interaction with more than one polymerase may stabilize it. FPC and Mcm10 significantly increased the processivity of the leading-strand complex, similar to that of L-Tag in the presence of RPA during DNA unwinding. FPC enhanced the L-Tag rate during leading-strand synthesis while maintaining its overall lifetime. Furthermore, FPC reduced the pausing time of leading-strand synthesis and stimulated its restart. Modulations in the processivity and rate of CMG were also observed in yeast (12). It is unknown how these kinetic modulations affect replisome activities during replication or other processes, such as chromatin remodeling, transcription, and repair.

The possibility of an explanation for FPC and Mcm10 participation in the SV40 replisome seems intriguing. Claspin binds to L-Tag during immunoprecipitation (54); however, Mcm10 does not colocalize with L-Tag foci *in vivo* (56). Our results cannot confirm if both FPC and Mcm10 or only one of them bind to the L-Tag at the fork. It is possible therefore that the lack of colocalization of Mcm10 and L-Tag *in vivo* may indicate that only FPC binds the L-Tag at the fork and that Mcm10 is recruited under DNA replication stress, e.g. transcription–replication conflicts (86). Nonetheless, although the Mcm10 and L-Tag foci are spatially uncorrelated *in vivo*, the punctate pattern of Mcm10 distribution indicates that a number of SV40 replisomes may still contain Mcm10 (56). Furthermore, if we assume that Mcm10 does not physically interact with L-Tag, Mcm10 could still functionally interact with L-Tag by binding the fork junction (33). In another possibility, it has been shown in yeast that diubiquitinated Mcm10 physically interacts with PCNA via a PIP box in Mcm10 (85). If this feature is conserved among eukaryotes, Mcm10 would be able to bind the SV40 replisome via PCNA.

SV40 replication responds to genotoxic and non-genotoxic stress. For example, Claspin regulates cellular DNA synthesis and repair in response to various stresses (87–93). TIMELESS and Tipin are involved in the molecular mechanism of UV-dependent checkpoint activation (32,94–96). Mcm10 inhibits fork regression through strand annealing (33). The interactions of FPC and Mcm10 with the SV40 replisome provided additional regulatory mechanisms, ensuring a more accurate bypass of DNA damage by modulating the replication rate and restarting, contradictory to error-prone DNA replication and fork collapse. Future studies must investigate how the SV40 replisome coordinates with FPC and Mcm10 *in vivo*, which may help develop molecularly targeted therapies against this broad spectrum of viruses and virus-related cancers.

## Supplementary Material

gkae565_Supplemental_File

## Data Availability

The data underlying this article are available in the article and in its online supplementary material.
