# Supplementary material for: Single-molecule characterization of SV40 replisome and novel factors: human FPC and Mcm10
Source: Nucleic Acids Res. 2024 Jul 5;52(15):8880–96. doi: 10.1093/nar/gkae565 (PMC11347169; doi:10.1093/nar/gkae565)
Supplement: gkae565_Supplemental_File [file gkae565_supplemental_file.pdf]

Supplementary Data

Single-Molecule Characterization of SV40 Replisome and Novel Factors: Human FPC and Mcm10

Yujing Ouyang, Amani Al-Amodi, Muhammad Tehseen, Lubna Alhudhali, Afnan Shirbini, Masateru Takahashi, Vlad-Stefan Raducanu, Gang Yi, Ammar Dan Azumi, Alfredo De Biasio, and Samir M. Hamdan

Includes:

Supplementary Table: 1

Supplementary Figures: 27

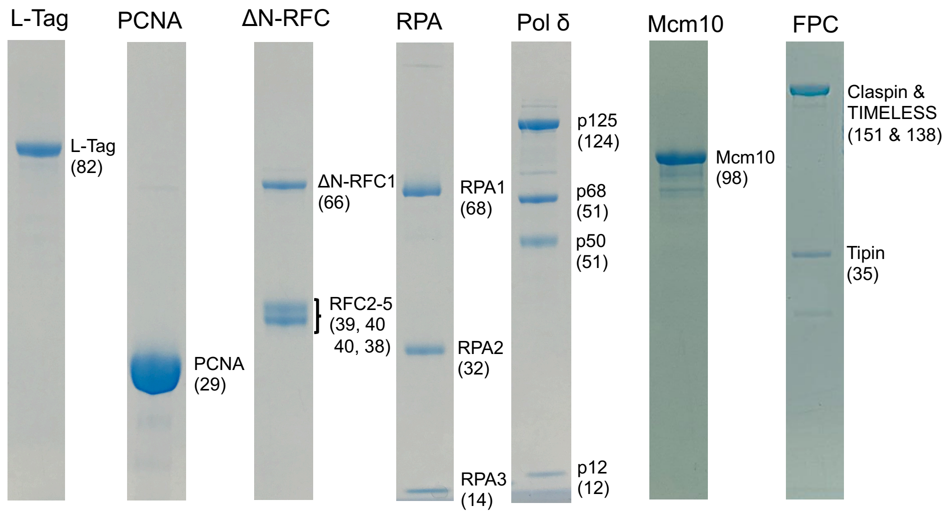

Figure S1. Purified SV40 replication proteins. Coomassie blue stained SDS-PAGE gel images of SV40 L-Tag, and human Proliferating Cell Nuclear Antigen (PCNA), Replication Factor C (RFC), Replication Protein A (RPA), Pol δ, mini-chromosome maintenance protein 10 (Mcm10), and fork protection complex (FPC). Individual lanes from gel images are shown with each subunit labeled with its molecular mass (kDa).

Table S1. Oligonucleotides mentioned in MATERIALS AND METHODS. Slash symbols contain modification types at the 5′ end.

Name	Sequence (5′-3′)
1	TGTCGGACTAGAGATCTGAGCAGTCCTAACTGGAAATCTAGCACTGTGGAG
2	/Cy5/TTTTTGGTTAGGACTGCTCAGATCTCTAGTCCGACA
3	TTTTTGGTTAGGACTGCTCAGATCTCTAGTCCGACA
4	/Digoxigenin/GAACAACAACCCGCAACATCCGCTTTCC
5	AGGGCCCGCAGAGTGGATGTTTGACATGG
6	AGGGCCCAATTCCGGCGTGAATGTTACTGAATC
7	GCTACACCATTGCGTGCATCGAGTAAGTCGC
8	/Biotin/AAAAAAAAAAAAAAAAGGTAGAGCGAAAGGTGACCAGAGCCAGTCTCG

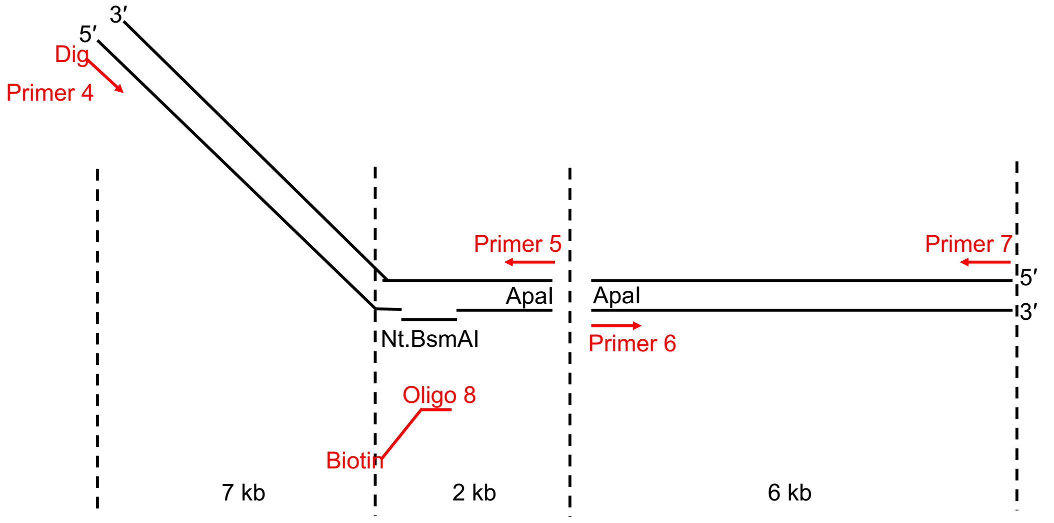

Figure S2. Schematic of the leading strand bead substrate and its preparation. Both 9-kb and 6-kb dsDNA fragments are PCR products of λ DNA, corresponding to separate regions. Nicking enzyme Nt.BsmAI opens phosphodiester bonds at the same strand of the 9-kb dsDNA, 7 kb from the Digoxigenin end and 2 kb from the other end. Excess oligo 8 competes the nicked original strand away, leaving a ssDNA gap to form a fork at the same time. The modified 9-kb fragment and the 6-kb fragment are digested by ApaI, annealed together through the sticky ends, and ligated. As a result, when the bead binds this substrate, it is on the leading-strand arm of the fork, 7 kb away from the fork junction.

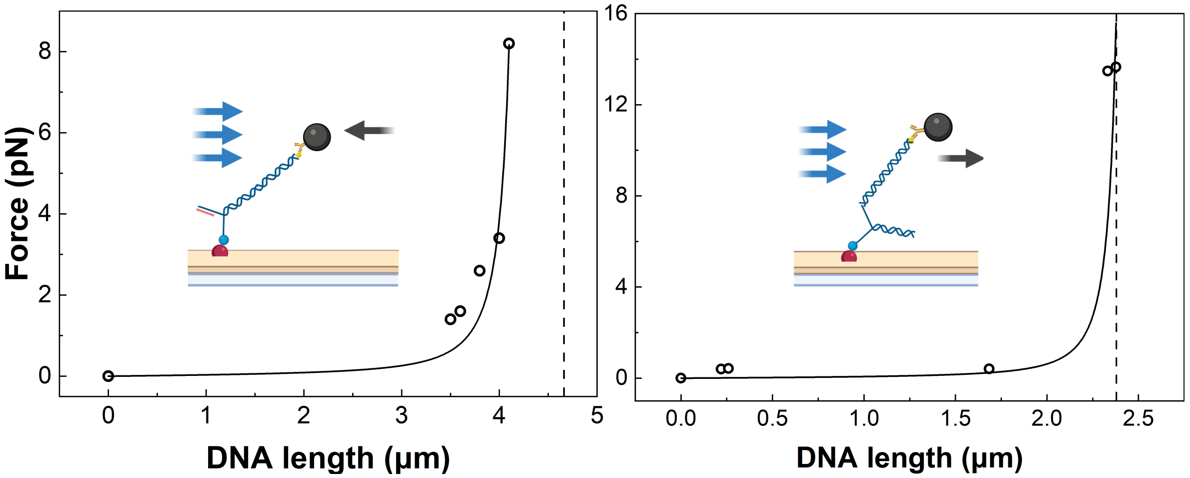

Figure S3. The force calibration plots of the 13.5-kb dsDNA substrate (left one) and the 7-kb leading strand bead substrate (right one), whose geometries are illustrated as the cartoons. The black lines represent worm-like chain (WLC) model fits. All schematics were created with BioRender.com.

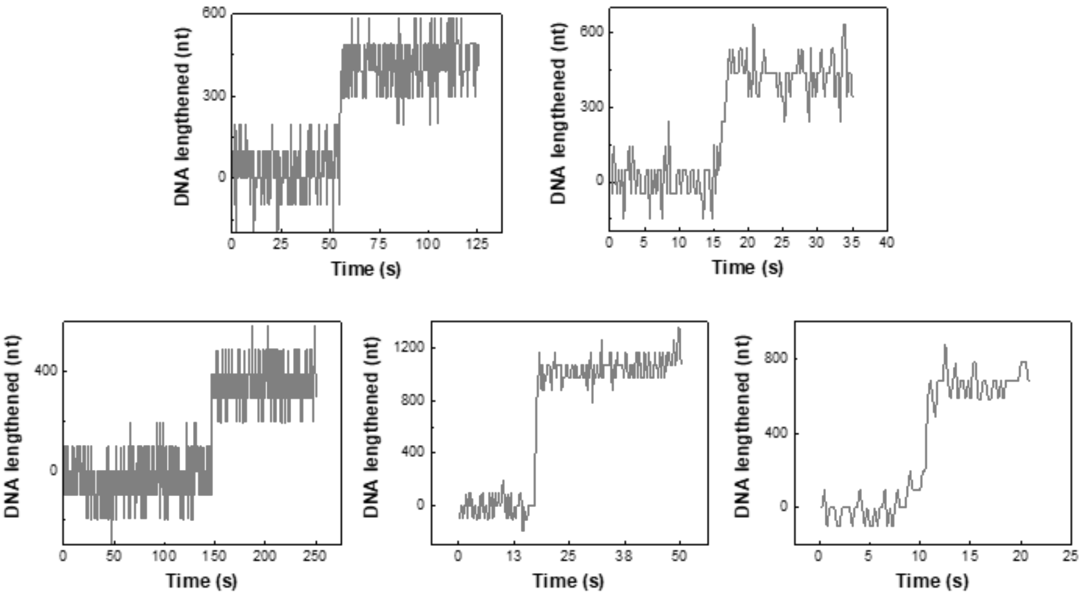

Figure S4. Representative traces showing Pol δ-dependent primer extension.

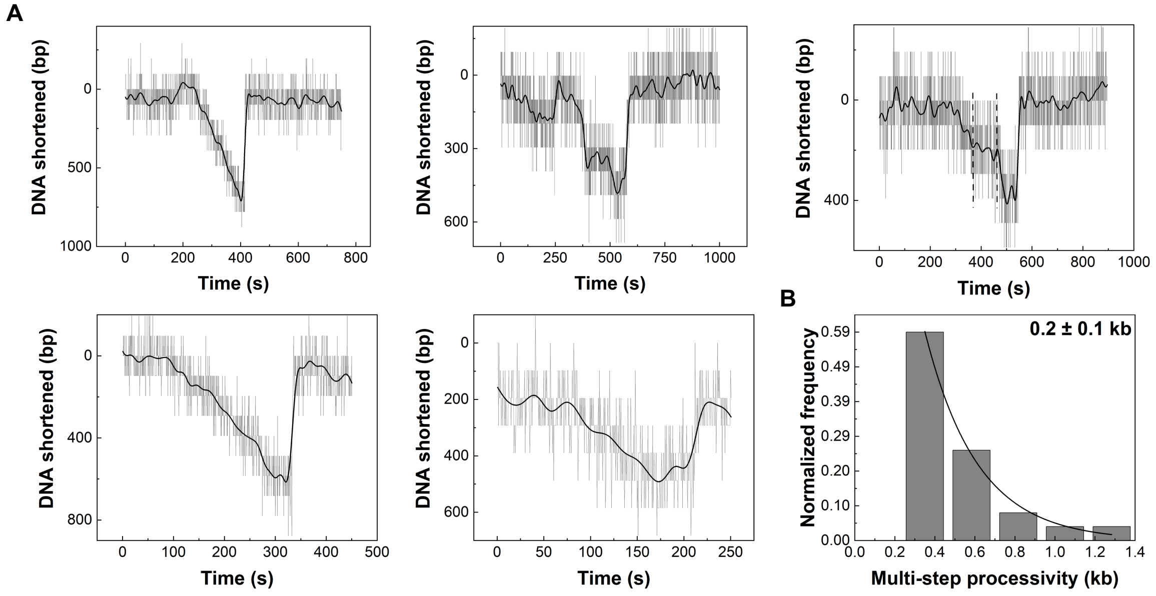

Figure S5. (**A**) Representative traces showing L-Tag-dependent unwinding. Black lines represent smoothing by Fast Fourier Transform (FFT) filters. Dashed lines indicate a pause. (**B**) Histogram of multi-step processivities of L-Tag unwinding. The black line represents a a single exponential decay fit with a processivity of 0.2 ± 0.1 kb (mean ± SEM) (n = 51 traces).

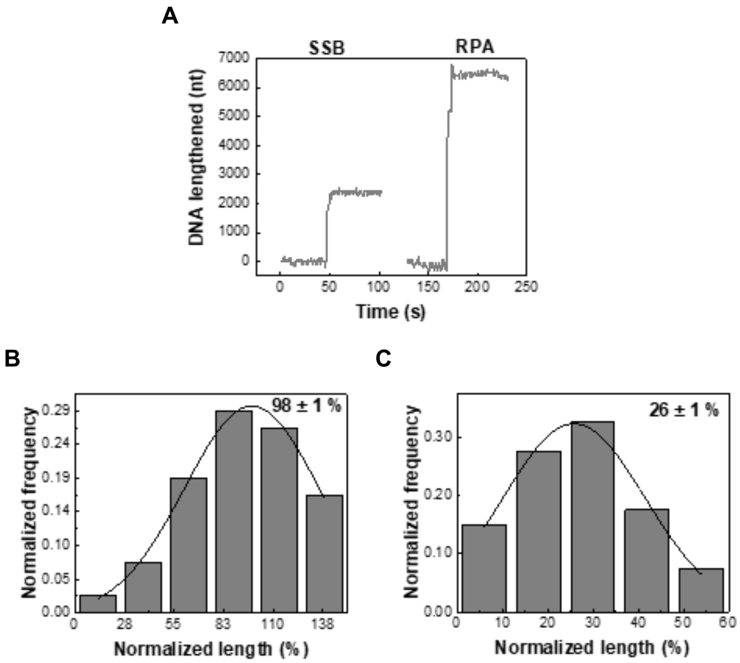

Figure S6. Conversion factors of ssDNA, coated with either *E. coli* SSB or human RPA. (**A**) Representative traces showing ssDNA-stretching effects of *E. coli* SSB and human RPA. (**B**) Ratio between full-length dsDNA substrate and human RPA lengthening for 42 DNA molecules. Mean ratio 98 ± 1 % (mean ± SEM). (**C**) Ratio between full-length dsDNA substrate and *E. coli* SSB lengthening for 40 DNA molecules. Mean ratio 26 ± 1 % (mean ± SEM). SSB, ssDNA-binding protein; RPA, Replication Protein A.

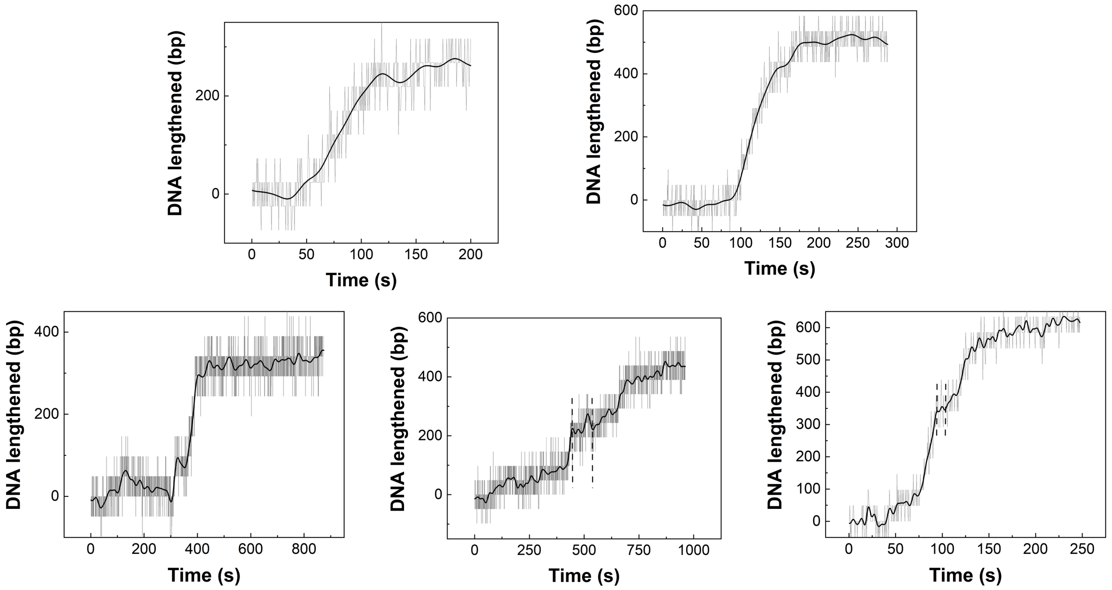

Figure S7. Representative traces showing L-Tag-dependent unwinding with Replication Protein A (RPA). Black lines represent smoothing by Fast Fourier Transform (FFT) filters. Dashed lines indicate pauses.

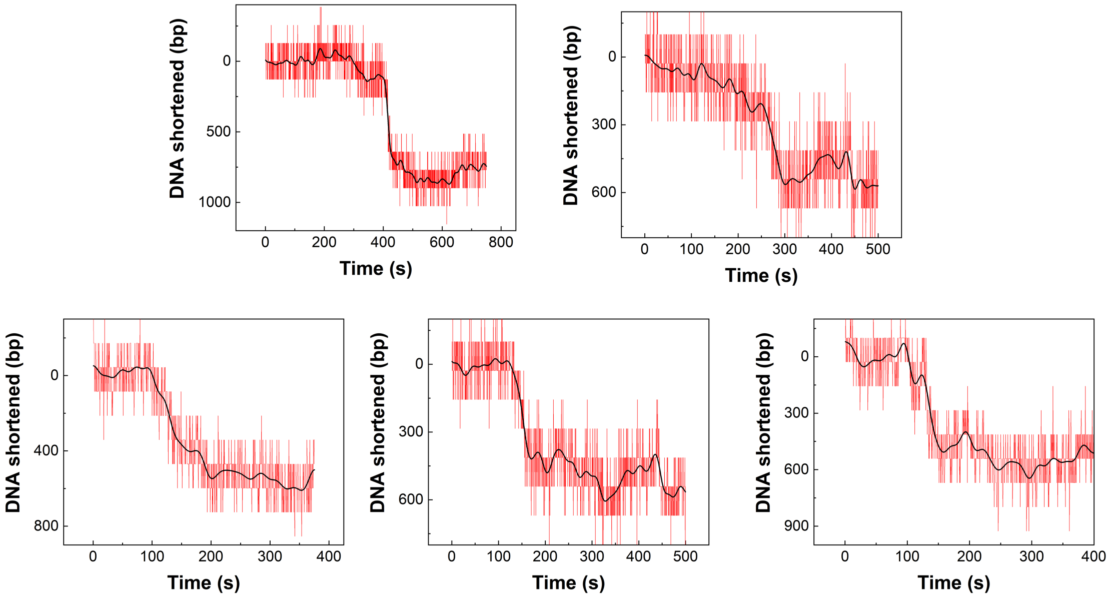

Figure S8. Representative traces showing SV40 replisome-dependent leading-strand synthesis with *E. coli* ssDNA-binding protein (SSB). Black lines represent smoothing by Fast Fourier Transform (FFT) filters.

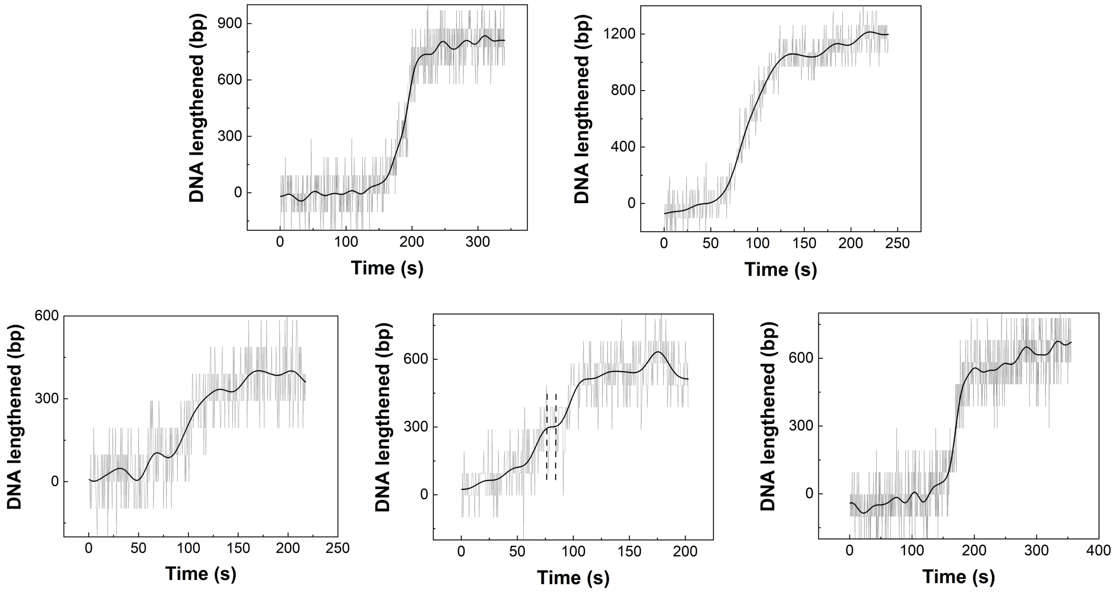

Figure S9. Representative trace showing SV40 replisome-dependent leading-strand synthesis with human Replication Protein A (RPA). Black lines represent smoothing by Fast Fourier Transform (FFT) filters. Dashed lines indicate a pause.

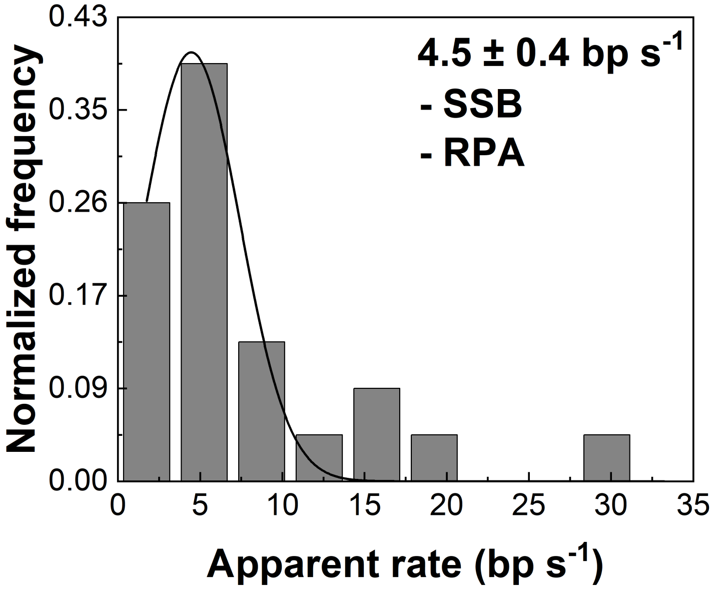

Figure S10. Histogram of SV40 leading-strand synthesis apparent rates without *E. coli* ssDNA-binding protein (SSB) or human Replication Protein A (RPA). The black line represents a Gaussian fit with a rate of 4.5 ± 0.4 bp/s (mean ± SEM) (n =23 events).

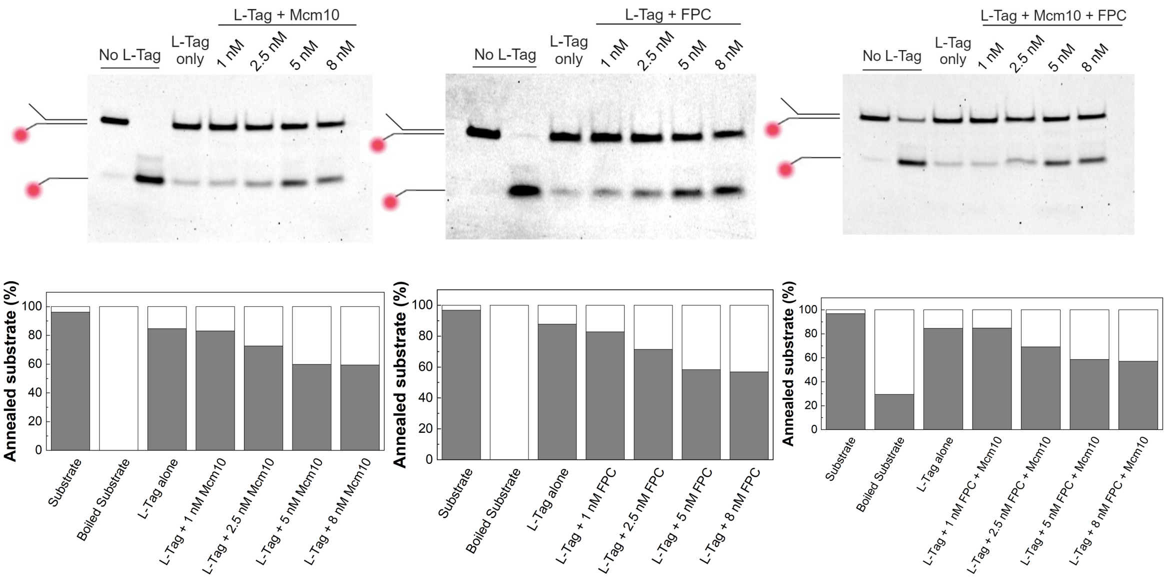

Figure S11. Fork protection complex (FPC) and mini-chromosome maintenance protein 10 (Mcm10) stimulate L-Tag unwinding and corresponding quantifications. Native PAGE gels of L-Tag unwinding products, 5′ Cy5-labelled. Reactions were performed as in Fig. 4, described in MATERIALS AND METHODS. All schematics and gel labelling were created with BioRender.com.

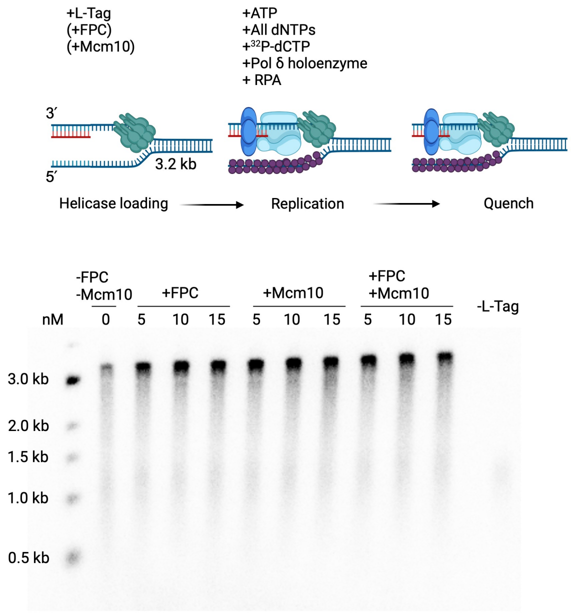

Figure S12. Fork protection complex (FPC) and mini-chromosome maintenance protein 10 (Mcm10) stimulate SV40 leading-strand synthesis. Schematic of SV40 leading-strand synthesis bulk assays and alkaline agarose gel of SV40 leading-strand synthesis products, ^{32}P-dCTP-labelled (nucleotide incorporation). Reactions were performed as described in MATERIALS AND METHODS. All schematics and gel labelling were created with BioRender.com.

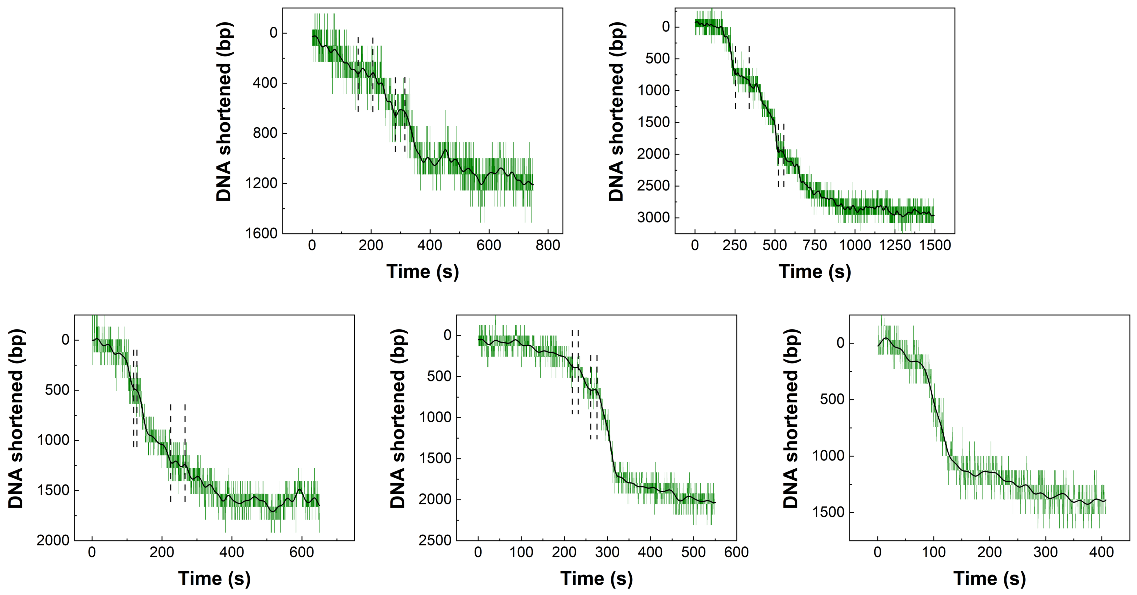

Figure S13. Representative traces showing SV40 replisome-dependent leading-strand synthesis with *E. coli* ssDNA-binding protein (SSB) and pre-assembled fork protection complex (FPC). Black lines represent smoothing by Fast Fourier Transform (FFT) filters. Dashed lines indicate pauses.

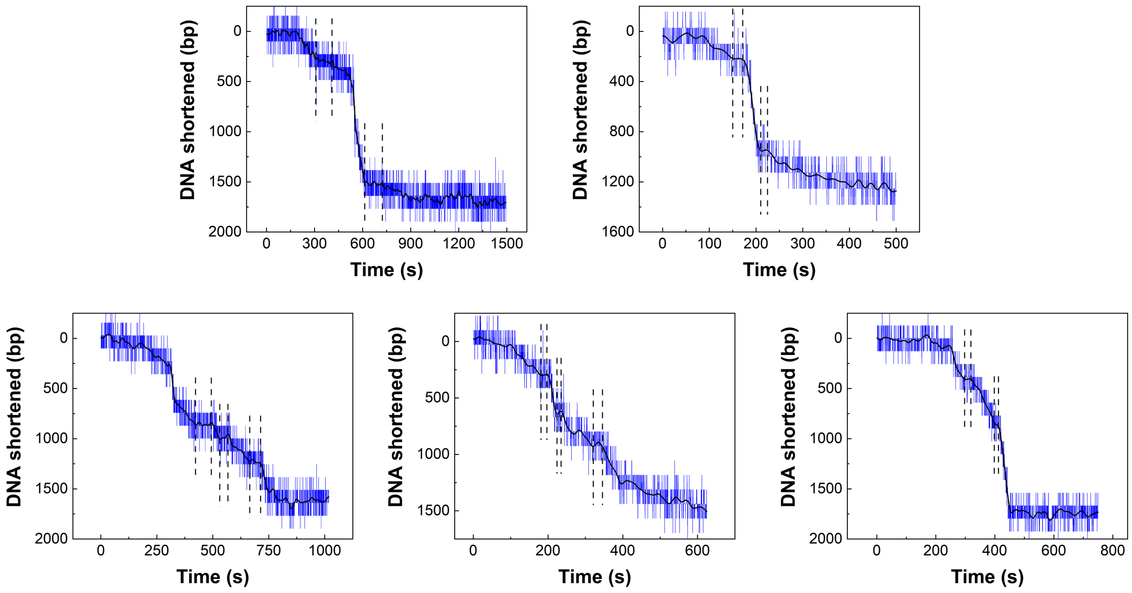

Figure S14. Representative traces showing SV40 replisome-dependent leading-strand synthesis with *E. coli* ssDNA-binding protein (SSB) and pre-assembled mini-chromosome maintenance protein 10 (Mcm10). Black lines represent smoothing by Fast Fourier Transform (FFT) filters. Dashed lines indicate pauses.

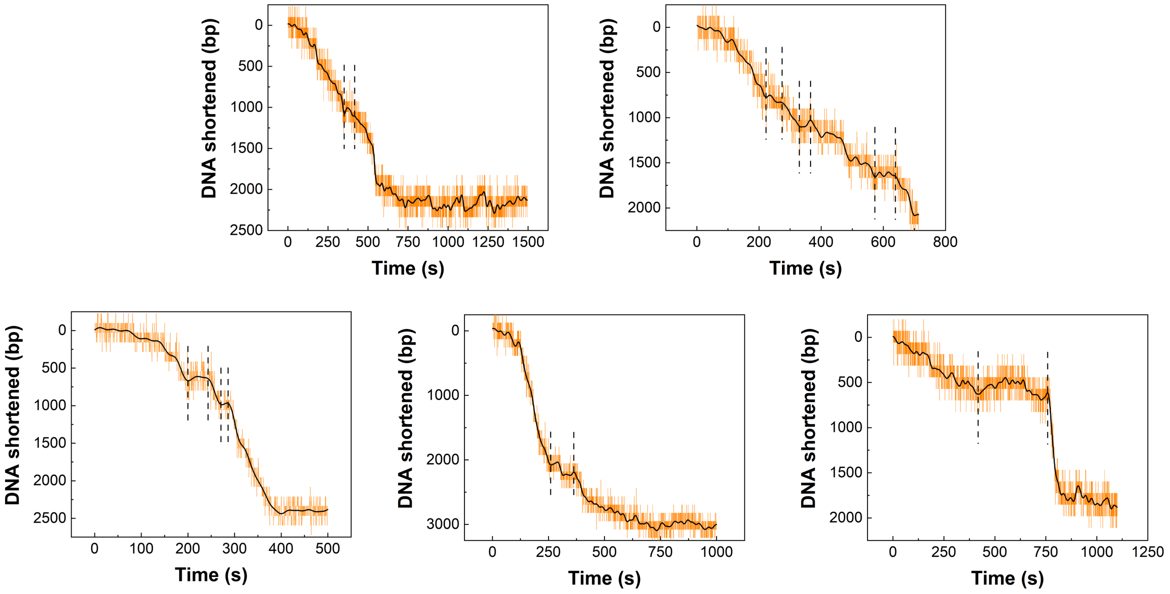

Figure S15. Representative traces showing SV40 replisome-dependent leading-strand synthesis with *E. coli* ssDNA-binding protein (SSB) and pre-assembled fork protection complex (FPC) and mini-chromosome maintenance protein 10 (Mcm10). Black lines represent smoothing by Fast Fourier Transform (FFT) filters. Dashed lines indicate pauses.

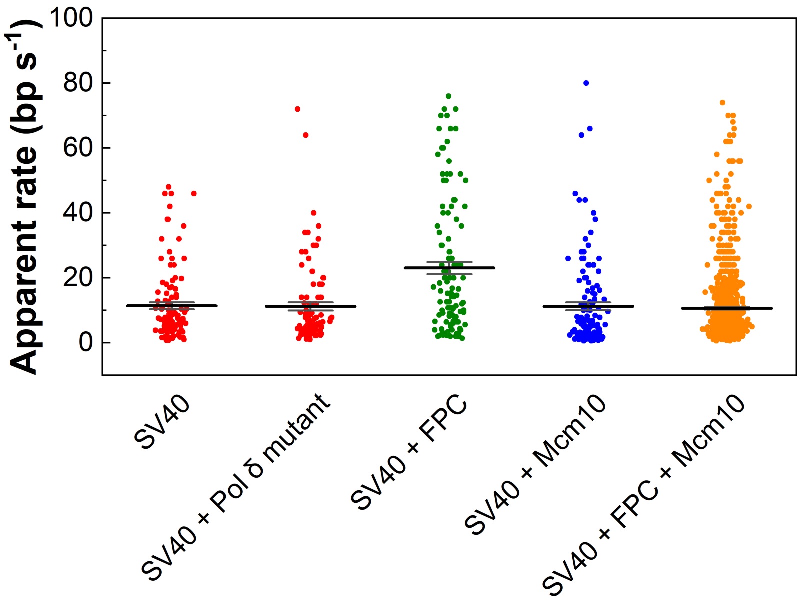

Figure S16. Scattered plots of single-molecule apparent rates. The black lines represent means. The grey whiskers and caps represent standard error of mean (SEM). The numbers of dots are the same as their sample sizes of histograms.

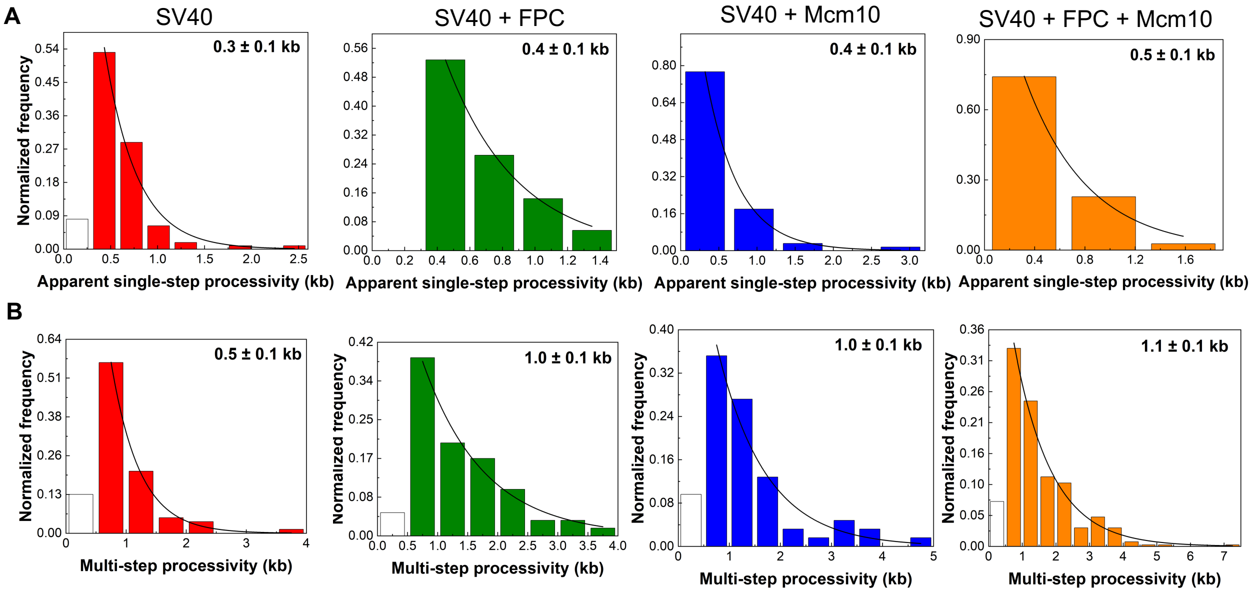

Figure S17. SV40 leading-strand synthesis processivity statistics. (**A**) For apparent single-step processivities, fitting SV40 alone with a single exponential decay (black line) gives a processivity of 0.3 ± 0.1 kb (mean ± SEM) (n = 111 traces). SV40 with FPC, 0.4 ± 0.1 kb (mean ± SEM) (n = 124 traces). SV40 with Mcm10, 0.4 ± 0.1 kb (mean ± SEM) (n = 128 traces). SV40 with FPC and Mcm10, 0.5 ± 0.1 kb (mean ± SEM) (n = 332 traces). (**B**) For multi-step processivities, fitting SV40 alone with a single exponential decay (black line) gives a processivity of 0.5 ± 0.1 kb (mean ± SEM) (n = 78 traces). SV40 with FPC, 1.0 ± 0.1 kb (mean ± SEM) (n = 59 traces). SV40 with Mcm10, 1.0 ± 0.1 kb (mean ± SEM) (n = 62 traces). SV40 with FPC and Mcm10, 1.1 ± 0.1 kb (mean ± SEM) (n = 386 traces). FPC, fork protection complex; Mcm10, mini-chromosome maintenance protein 10.

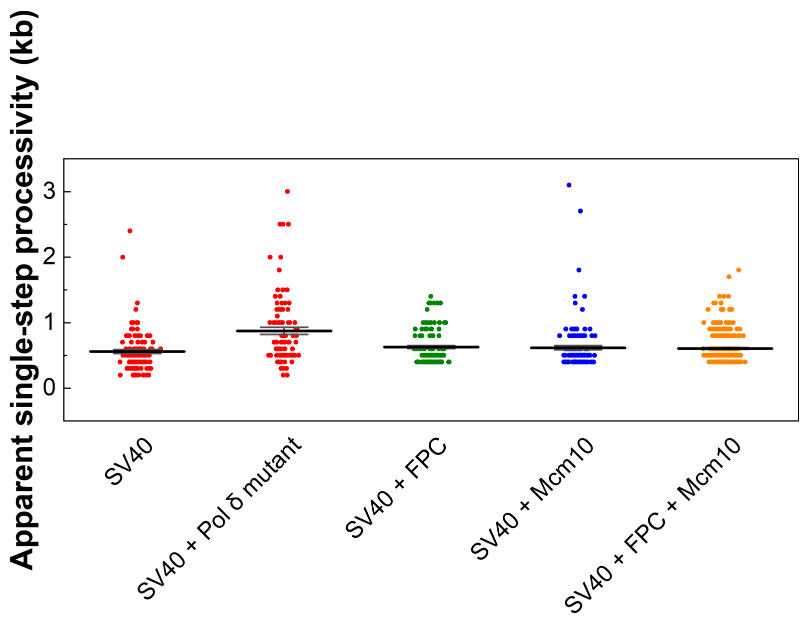

Figure S18. Scattered plots of single-molecule apparent single-step processivities. The black lines represent means. The grey whiskers and caps represent standard error of mean (SEM). The numbers of dots are the same as their sample sizes of histograms.

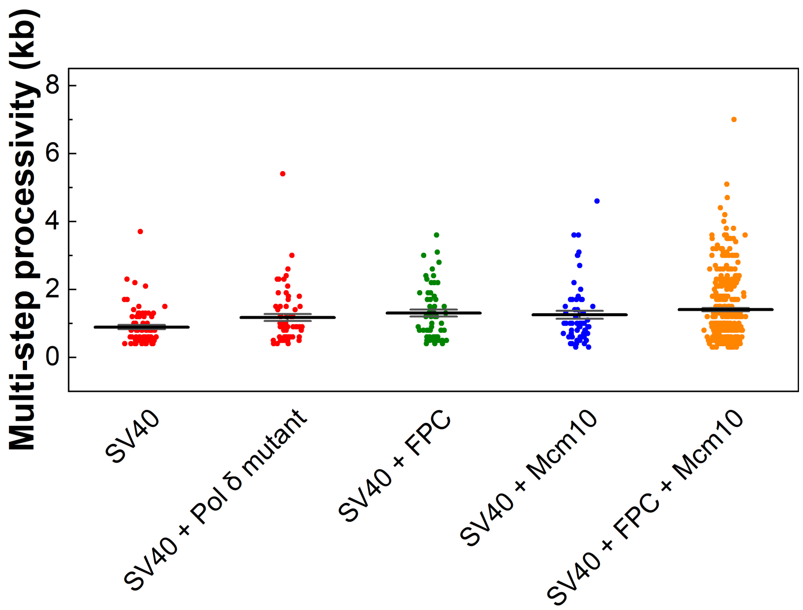

Figure S19. Scattered plots of single-molecule multi-step processivities. The black lines represent means. The grey whiskers and caps represent standard error of mean (SEM). The numbers of dots are the same as their sample sizes of histograms.

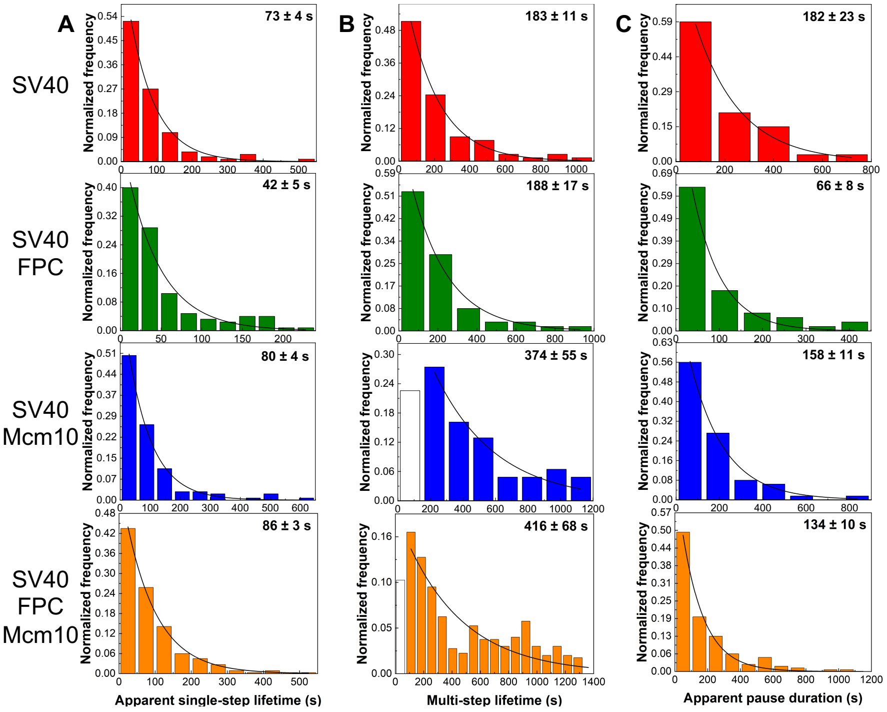

Figure S20. SV40 leading-strand synthesis lifetime and pause duration statistics. (**A**) For the apparent single-step lifetimes, fitting SV40 alone with a single exponential decay (black line) gives a lifetime of 73 ± 4 s (mean ± SEM) (n = 111 traces). SV40 with FPC, 42 ± 5 s (mean ± SEM) (n = 124 traces). SV40 with Mcm10, 80 ± 4 s (mean ± SEM) (n = 136 traces). SV40 with FPC and Mcm10, 86 ± 3 s (mean ± SEM) (n = 331 traces). (**B**) For the multi-step lifetimes, fitting SV40 alone with a single exponential decay (black line) gives a lifetime of 183 ± 11 s (mean ± SEM) (n = 78 traces). SV40 with FPC, 188 ± 17 s (mean ± SEM) (n = 59 traces). SV40 with Mcm10, 374 ± 55 s (mean ± SEM) (n = 62 traces). SV40 with FPC and Mcm10, 416 ± 68 s (mean ± SEM) (n = 386 traces). (**C**) For the apparent pause durations, fitting SV40 alone with a single exponential decay (black line) gives a pause duration of 182 ± 23 s (mean ± SEM) (n = 34 traces). SV40 with FPC, 66 ± 8 s (mean ± SEM) (n = 51 traces). SV40 with Mcm10, 158 ± 11 s (mean ± SEM) (n = 63 traces). SV40 with FPC and Mcm10, 134 ± 10 s (mean ± SEM) (n = 159 traces). FPC, fork protection complex; Mcm10, mini-chromosome maintenance protein 10.

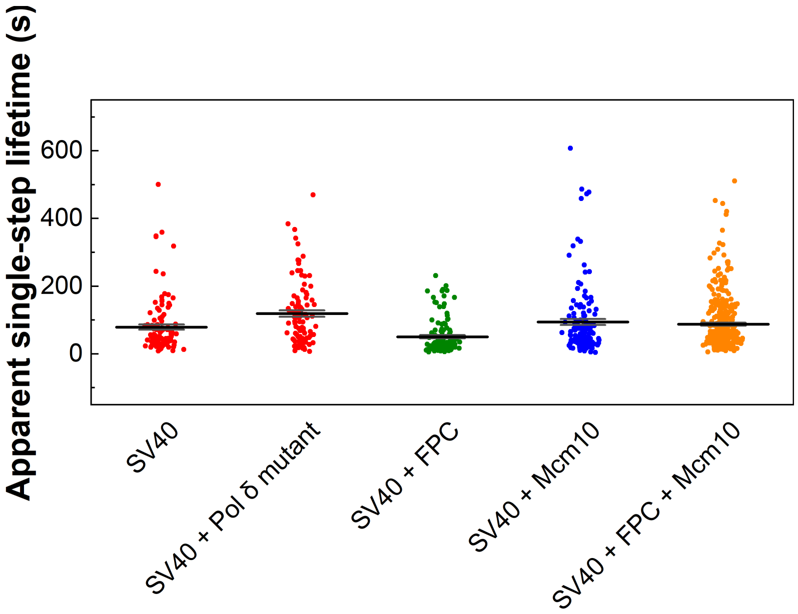

Figure S21. Scattered plots of single-molecule apparent single-step lifetimes. The black lines represent means. The grey whiskers and caps represent standard error of mean (SEM). The numbers of dots are the same as their sample sizes of histograms.

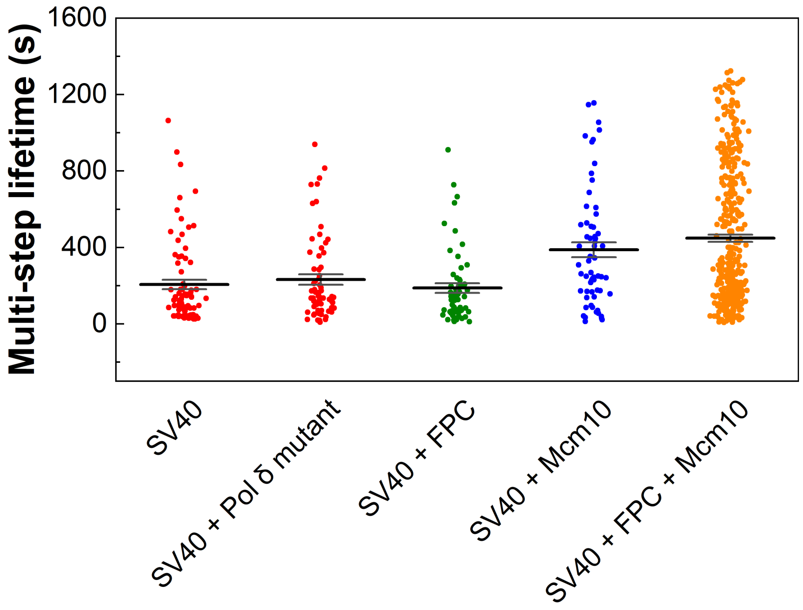

Figure S22. Scattered plots of single-molecule multi-step lifetimes. The black lines represent means. The grey whiskers and caps represent standard error of mean (SEM). The numbers of dots are the same as their sample sizes of histograms.

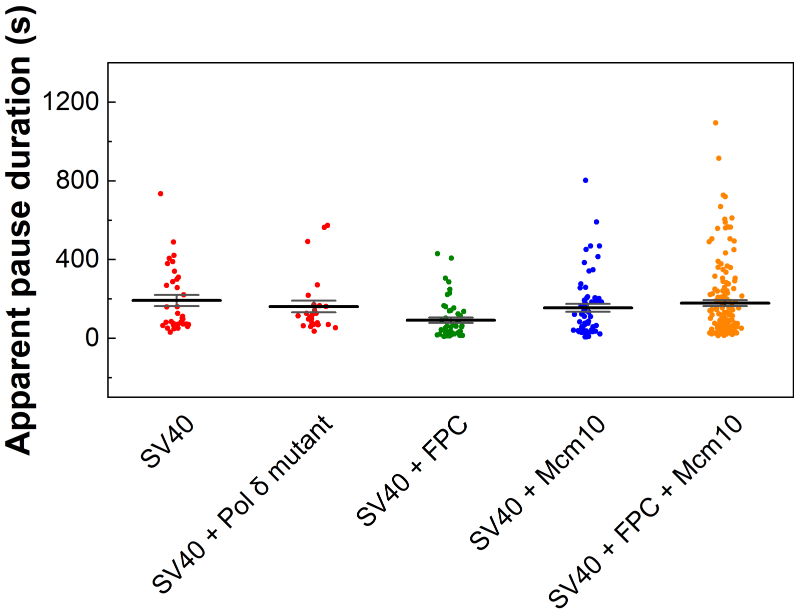

Figure S23. Scattered plots of single-molecule apparent pause durations. The black lines represent means. The grey whiskers and caps represent standard error of mean (SEM). The numbers of dots are the same as their sample sizes of histograms.

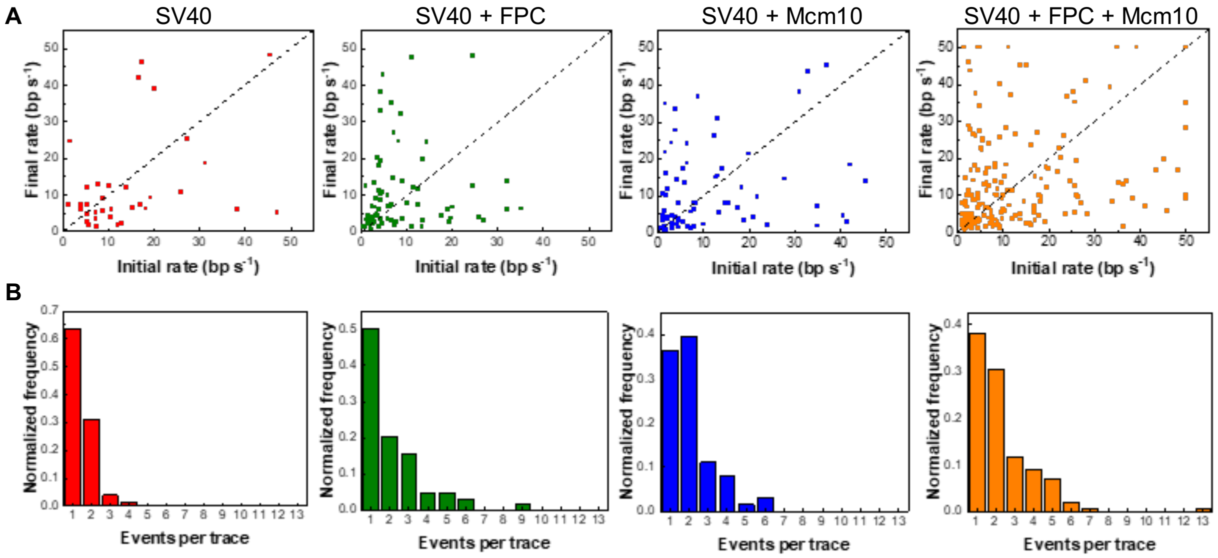

Figure S24. SV40 leading-strand synthesis rate fluctuation. (**A**) Transition plots of SV40 leading-strand synthesis rates with multiple events on the same traces. For SV40 alone, the distance from the diagonal (dashed line) is 6.8 ± 1.2 bp/s (mean ± SEM). For SV40 with FPC, the distance from the diagonal (dashed line) is 6.7 ± 0.8 bp/s (mean ± SEM). For SV40 with Mcm10, the distance from the diagonal (dashed line) is 7.2 ± 0.9 bp/s (mean ± SEM). For SV40 with FPC and Mcm10, the distance from the diagonal (dashed line) is 7.7 ± 0.6 bp/s (mean ± SEM). (**B**) The number of events per trace of SV40 leading-strand synthesis. FPC, fork protection complex; Mcm10, mini-chromosome maintenance protein 10.

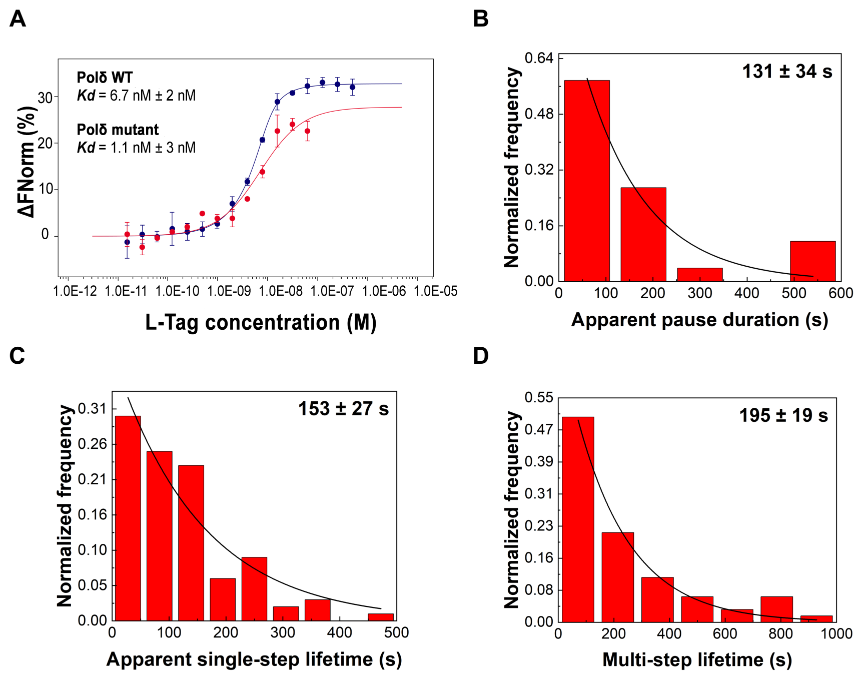

Figure S25. Microscale thermophoresis (MST) between L-Tag and Pol δ and polymerase exchange statistics. (**A**) L-Tag-Pol δ interactions in MST. The K_d of L-Tag-WT Pol δ interaction is 6.7 ± 2 nM. The K_d of L-Tag-Pol δ polymerase-deficient mutant interaction is 1.1 ± 3 nM. (**B**) Histograms of SV40 leading-strand synthesis apparent pause durations. The black line represents a single exponential decay fit with a pause duration of 131 ± 34 s (mean ± SEM) (n = 26 traces). (**C**) Histogram of SV40 leading-strand synthesis apparent single-step lifetimes. The black line represents a single exponential decay fit with a lifetime of 153 ± 27 s (mean ± SEM) (n = 96 events). (**D**) Histogram of SV40 leading-strand synthesis multi-step lifetimes. The black line represents a single exponential decay fit with a lifetime of 195 ± 19 s (mean ± SEM) (n = 64 traces).

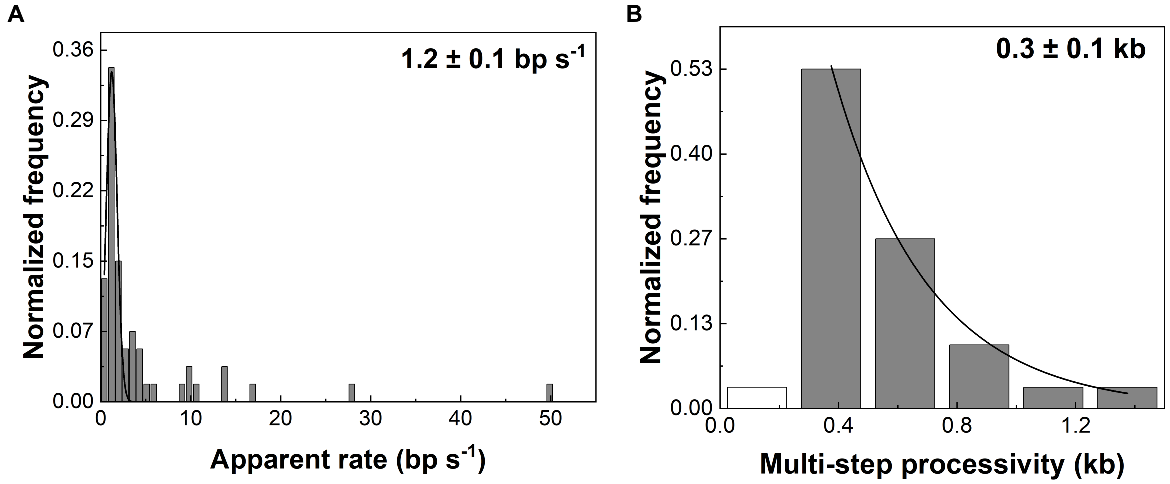

Figure S26. Histograms of L-Tag unwinding with the addition of Pol δ polymerase-and-exonuclease-deficient mutant. (**A**) Histograms of L-Tag unwinding rates. The black line represents a Gaussian fit with a rate of 1.2 ± 0.1 bp/s (mean ± SEM) (n = 55 events). (**B**) Histograms of L-Tag unwinding multi-step processivities. The black line represents a single-exponential decay fit with a processivity of 0.3 ± 0.1 kb (mean ± SEM) (n = 30 traces).

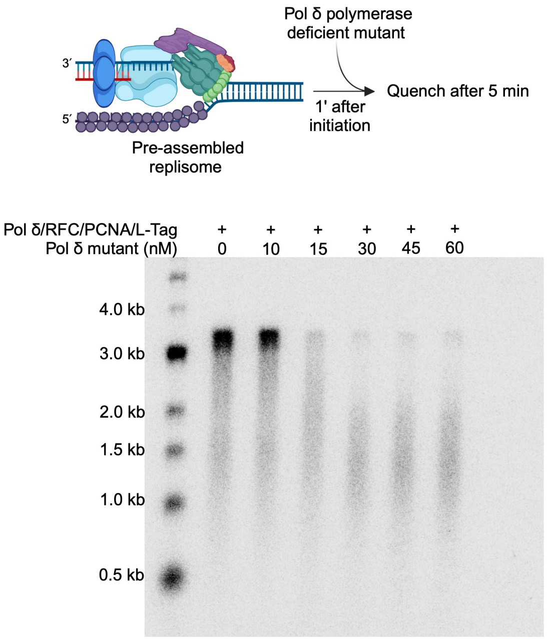

Figure S27. Pol δ polymerase-deficient mutant terminates SV40 leading-strand synthesis after certain processivity, Pol δ polymerase-deficient mutant concentration titration. Schematic of Pol δ polymerase-deficient mutant exchange bulk assays and alkaline agarose gels of SV40 leading-strand synthesis products, ^{32}P-dCTP-labelled (nucleotide incorporation). Reactions were performed as described in MATERIALS AND METHODS. All schematics and gel labelling were created with BioRender.com.

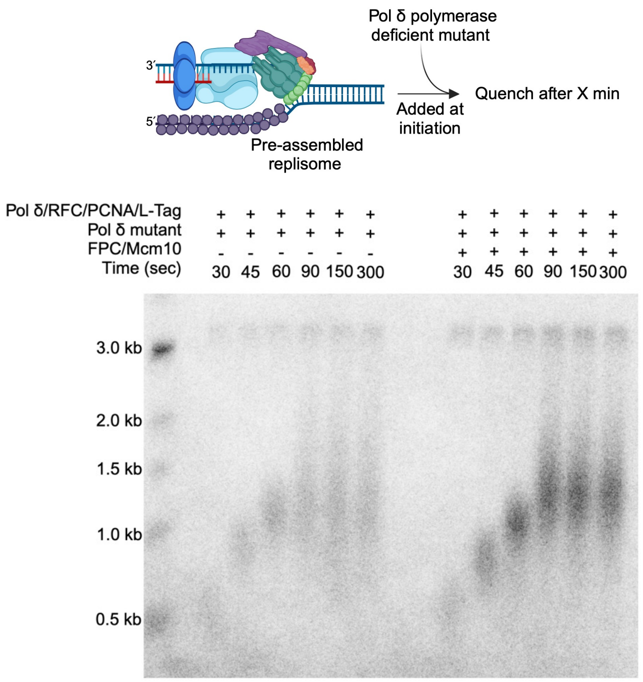

Figure S28. Pol δ polymerase-deficient mutant terminates SV40 leading-strand synthesis after certain processivity, time titration, with or without FPC and Mcm10. Schematic of Pol δ polymerase-deficient mutant exchange bulk assays and alkaline agarose gels of SV40 leading-strand synthesis products, ^{32}P-dCTP-labelled (nucleotide incorporation). Reactions were performed as described in MATERIALS AND METHODS. All schematics and gel labelling were created with BioRender.com.
